# *TP53* mutations and *TET2* deficiency cooperate to drive leukemogenesis and establish an immunosuppressive environment

**DOI:** 10.1172/JCI184021

**Published:** 2025-03-20

**Authors:** Pu Zhang, Ethan C. Whipp, Sarah J. Skuli, Mehdi Gharghabi, Caner Saygin, Steven A. Sher, Martin Carroll, Xiangyu Pan, Eric D. Eisenmann, Tzung-Huei Lai, Bonnie K. Harrington, Wing Keung Chan, Youssef Youssef, Bingyi Chen, Alex Penson, Alexander M. Lewis, Cynthia R. Castro, Nina Fox, Ali Cihan, Jean-Benoit Le Luduec, Susan DeWolf, Tierney Kauffman, Alice S. Mims, Daniel Canfield, Hannah Phillips, Katie E. Williams, Jami Shaffer, Arletta Lozanski, Tzyy-Jye Doong, Gerard Lozanski, Charlene Mao, Christopher J. Walker, James S. Blachly, Anthony F. Daniyan, Lapo Alinari, Robert A. Baiocchi, Yiping Yang, Nicole R. Grieselhuber, Moray J. Campbell, Sharyn D. Baker, Bradley W. Blaser, Omar Abdel-Wahab, Rosa Lapalombella

**Affiliations:** 1 Division of Hematology, Department of Internal Medicine, The Ohio State University, Columbus, Ohio, USA.; 2 Molecular Pharmacology Program, Sloan Kettering Institute, New York, New York, USA.; 3 Division of Pharmaceutics and Pharmacology, College of Pharmacy, The Ohio State University, Columbus, Ohio, USA.; 4 University of Pennsylvania, Philadelphia, Pennsylvania, USA.; 5 Section of Hematology/Oncology, University of Chicago, Chicago, Illinois, USA.; 6 Joan and Sanford I. Weill Department of Medicine, Weill Cornell Medicine, New York, New York, USA.; 7 College of Veterinary Medicine, Michigan State University, East Lansing, Michigan, USA.; 8 Memorial Sloan Kettering Cancer Center, New York, New York, USA.; 9 Leukemia Research Program, The Ohio State University James Comprehensive Cancer Center, Columbus, Ohio, USA.; 10 Division of Cancer Biology, Cedars Sinai Medical Center, Los Angeles, California, USA.

**Keywords:** Hematology, Inflammation, Oncology, Leukemias, Mouse models, p53

## Abstract

Mutations and deletions in *TP53* are associated with adverse outcomes in patients with myeloid malignancies, and there is an urgent need for the development of improved therapies for *TP53*-mutant leukemias. Here, we identified mutations in *TET2* as the most common co-occurring mutation in patients with *TP53-*mutant acute myeloid leukemia (AML). In mice, combined hematopoietic-specific deletion of *TET2* and *TP53* resulted in enhanced self-renewal compared with deletion of either gene alone. *Tp53/Tet2* double-KO mice developed serially transplantable AML. Both mice and patients with AML with combined *TET2/TP53* alterations upregulated innate immune signaling in malignant granulocyte-monocyte progenitors, which had leukemia-initiating capacity. A20 governs the leukemic maintenance by triggering aberrant noncanonical NF-κB signaling. Mice with *Tp53/Tet2* loss had expansion of monocytic myeloid-derived suppressor cells (MDSCs), which impaired T cell proliferation and activation. Moreover, mice and patients with AML with combined *TP53/TET2* alterations displayed increased expression of the TIGIT ligand, CD155, on malignant cells. TIGIT-blocking antibodies augmented NK cell–mediated killing of *Tp53/Tet2* double-mutant AML cells, reduced leukemic burden, and prolonged survival in *Tp53/Tet2* double-KO mice. These findings describe a leukemia-promoting link between *TET2* and *TP53* mutations and highlight therapeutic strategies to overcome the immunosuppressive bone marrow environment in this adverse subtype of AML.

## Abstract 2

<blockquote type=”cite”> <div> <div> <div> Combined TET2 and TP53 loss collaborate to promote acute myeloid leukemia development and generate a targetable vulnerability through upregulation of TIGIT ligands on malignant cells. </div> </div> </div> </blockquote>

## Graphical abstract

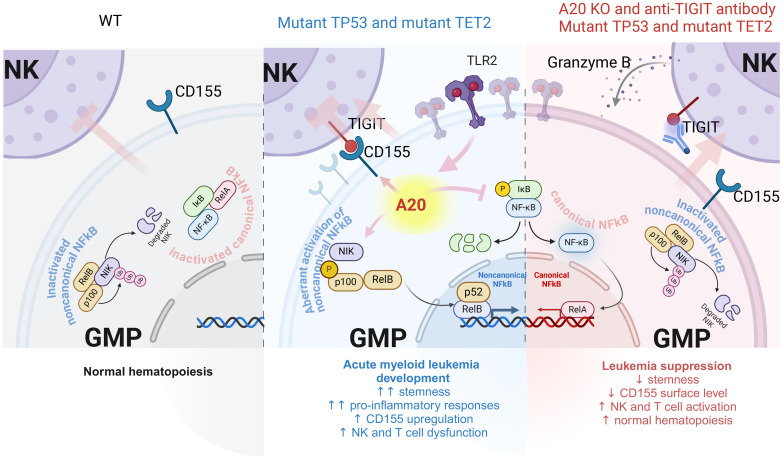

## Introduction

Mutations in *TP53* are independently associated with inferior survival in patients with myelodysplastic syndrome (MDS) and acute myeloid leukemia (AML) (1). *TP53* mutations are present in 10%–15% of patients with AML, frequently co-occur with complex karyotype, and are more common in patients with secondary or therapy-related AML. Mutations in *TP53* may occur years to decades before development of overt neoplasia and define a high-risk subtype of clonal hematopoiesis.

Several prior studies have evaluated genomic alterations that coexist with *TP53*-mutant myeloid neoplasms. Among these, loss of heterozygosity and deletion of the p53 locus on chromosome 17p are well described to co-occur with *TP53* mutations (1–5). Beyond additional alterations to *TP53*, combined mutations in *TP53* and *TET2* are observed in MDS and are associated with high risk of AML transformation (6, 7). Moreover, *TET2/TP53* mutation is an independent predictor of inferior survival after stem cell transplantation (8).

Given the profound adverse implications of *TP53* mutations in myeloid neoplasms, there is an urgent need for targeted therapies to treat *TP53*-mutant leukemias and prevent the progression of these high-risk malignancies. Due to the impact of *TP53* mutations on impairment of DNA damage response and induction of apoptosis there has been great hope that nontumor cell autonomous therapeutic strategies harnessing the immune system could be used to treat *TP53*-mutant leukemias. To this end, prior work has suggested that *TP53-*mutant MDS may be characterized by impairments in the function and abundance of a broad range of adaptive immune cells (9). In parallel, several studies have also documented increased expression of the immune checkpoints PD-1, PD-L1, and CTLA-4 on myeloid malignant cells in MDS and AML (9). Despite these observations, blockade of these specific immune checkpoints in MDS has failed to result in therapeutic benefit.

Herein, by studying two cohorts of patients with AML from different institutions, we found that 10% of *TP53*-mutant AML cases carry a concurrent *TET2* mutation and are associated with extremely poor clinical outcomes. Our findings showed that mice with combined deletion of *TET2* and *TP53* developed aggressive acute leukemia that closely resembled human AML. Functional analyses of patients with *TET2/TP53* comutant AML revealed activation of proinflammatory A20-mediated innate immune signaling in the malignant cells. We found clear evidence of T cell exhaustion, with upregulation of the TIGIT ligand, CD155, on malignant cells, both in mouse models and patients with AML harboring these comutations. Importantly, therapeutic blockade of the CD155-TIGIT axis with anti-TIGIT antibodies markedly promoted NK cell–mediated killing of *TP53/TET2* comutant AML cells and extended survival in mice engrafted with *Tp53/Tet2* double-KO AML cells. These findings provide evidence that *TP53* deficiency and *TET2* loss cooperate to drive the development of AML and that the immunosuppressive microenvironment seen in such cases may be amenable to therapeutic blockade of TIGIT signaling.

## Results

### Mutations in TET2 are frequent in TP53-mutant AML.

**Figure 1:**
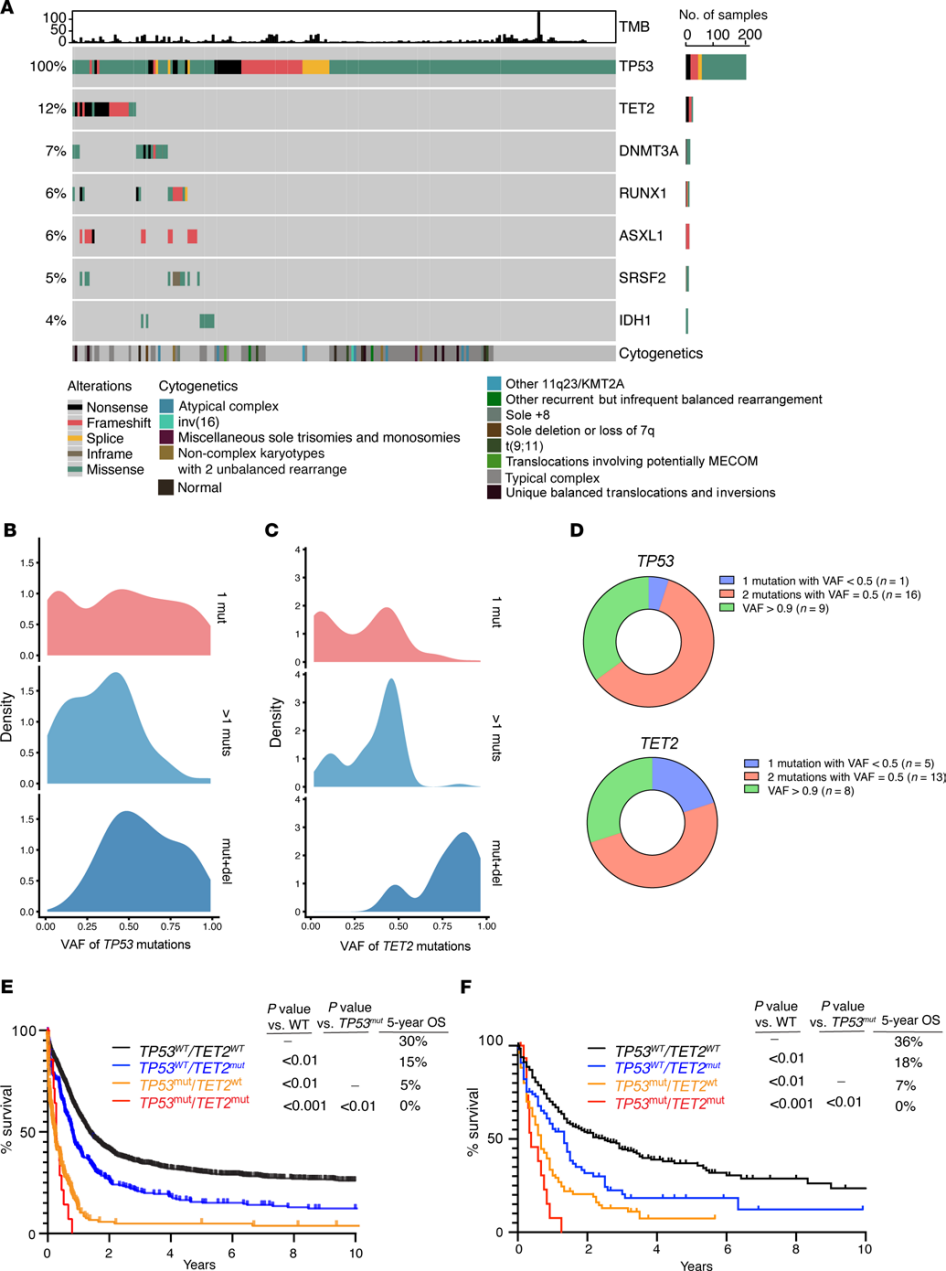
*TET2* mutations are common in *TP53*-mutant AML and confer an inferior outcome. (**A**) Oncoprint of patients with *TP53*-mutant AML with indication of the top 6 most common co-occurring genetic events as well as cytogenetics and tumor mutational burden (TMB) among 216 patients with AML with somatic *TP53* mutations. Data are from Alliance (our unpublished observations), Beat AML (10), and Rodriguez-Meira et al. (11). (**B**) Density estimation of variant allele frequency (VAF) of *TP53* mutations across 668 *TP53*-mutant patients (subdivided by patients with 1 *TP53* mutation, >1 *TP53* mutation, or *TP53* mutation plus deletion). Data are from AACR Project GENIE (12). (**C**) As in **B** but for *TET2* mutations from 80 *TP53*-mutant patients (subdivided by patients with 1 *TET2* mutation, >1 *TET2* mutation, or *TET2* mutation plus deletion). (**D**) The portion and VAF of *TET2* and *TP53* mutations in 26 patients with *TP53/TET2* comutations. Data are from Alliance, Beat AML (10), and Rodriguez-Meira et al. (11). (**E**) Kaplan-Meier survival curve in 1,603 patients with AML based on *TP53* and *TET2* mutational status from Alliance. OS, overall survival. (**F**) As in **E** but for an independent cohort of 653 patients with AML from the University of Chicago. A log-rank test was used for survival statistics.

We analyzed mutational data from 983 adult patients with *TP53*-mutant AML (216 from the Alliance for Clinical Trials in Oncology [referred to herein as Alliance]), the Beat AML program (10), and Rodriguez-Meira et al. (11) datasets, 99 from the University of Chicago, and 668 from American Association for Cancer Research (AACR) Project Genomics Evidence Neoplasia Information Exchange (GENIE) (12) to identify genetic alterations coexisting with *TP53* mutations in AML. The spectrum of *TP53* mutations in these cohorts is consistent with that in prior publications, with 50% consisting of a single nucleotide variant at hot spots R273, R248, H179, R175, and Y200 and the rest of the mutations being frameshift (28%) and nonsense (13%) mutations predicted to result in loss of function (Supplemental Figure 1A; supplemental material available online with this article; https://doi.org/10.1172/JCI184021DS1). Interestingly, the single most commonly comutated gene with *TP53* was *TET2*, with 12% of patients with *TP53*-mutant AML having a coexisting *TET2* mutation (Figure 1A and Supplemental Figure 1B). *TET2* mutations occurred along the entire open-reading frame with a few notable hot spots at R550, R1261, and I1873, as previously reported (13) (Supplemental Figure 1C).

Prior studies have highlighted that the allelic state of *TP53* is critical in determining prognosis in *TP53*-mutant MDS (13). While monoallelic *TP53* mutations were mostly subclonal, biallelic *TP53* mutations or *TP53* mutations accompanied by deletions of the remaining allele were clonal (Figure 1B). Similarly, individual *TET2* mutations in *TP53*-mutant AML were subclonal with a variant allele frequency (VAF) of 0.02, but biallelic *TET2* mutations or mutations plus deletions in *TET2* resulted in much higher VAFs and clonal dominance (Figure 1C). The spectrum of *TP53* and *TET2* mutations was similar along allelic states and had the same characteristic hot spot mutations (Supplemental Figure 1, A and C). Of these 26 cases with *TP53* and *TET2* comutations, 16 of them harbored 2 distinct *TP53* mutations with VAFs around 0.5; 9 had mutations with VAFs close to 1; 1 of them had a single mutation with VAF around 0.5 or less (Figure 1D). The frequency of multihit *TET2* mutations was much higher than that of single-hit *TET2* mutations. Homozygous biallelic mutations (VAF > 0.9) and heterozygous biallelic mutations (2 mutations with VAF = 0.5) account for 80% of all *TET2* mutation types. Biallelic mutation in *TET2* was more frequent compared with monoallelic mutation in the multihit *TP53* subgroup.

While the presence of *TP53* mutations was associated with poor survival, patients harboring concurrent *TP53* and *TET2* mutations exhibited even shorter overall survival compared with *TP53* or *TET2* single-mutated patients in both the Alliance and University of Chicago datasets (*P* < 0.01) (Figure 1, E and F). Altogether, these data identify the frequent co-occurrence of *TET2* mutations among *TP53*-mutant AML and suggest functional importance of loss of *TET2* in this adverse prognostic group of patients with AML.

### Loss of Tet2 and Tp53 expression results in lethal AML.

**Figure 2:**
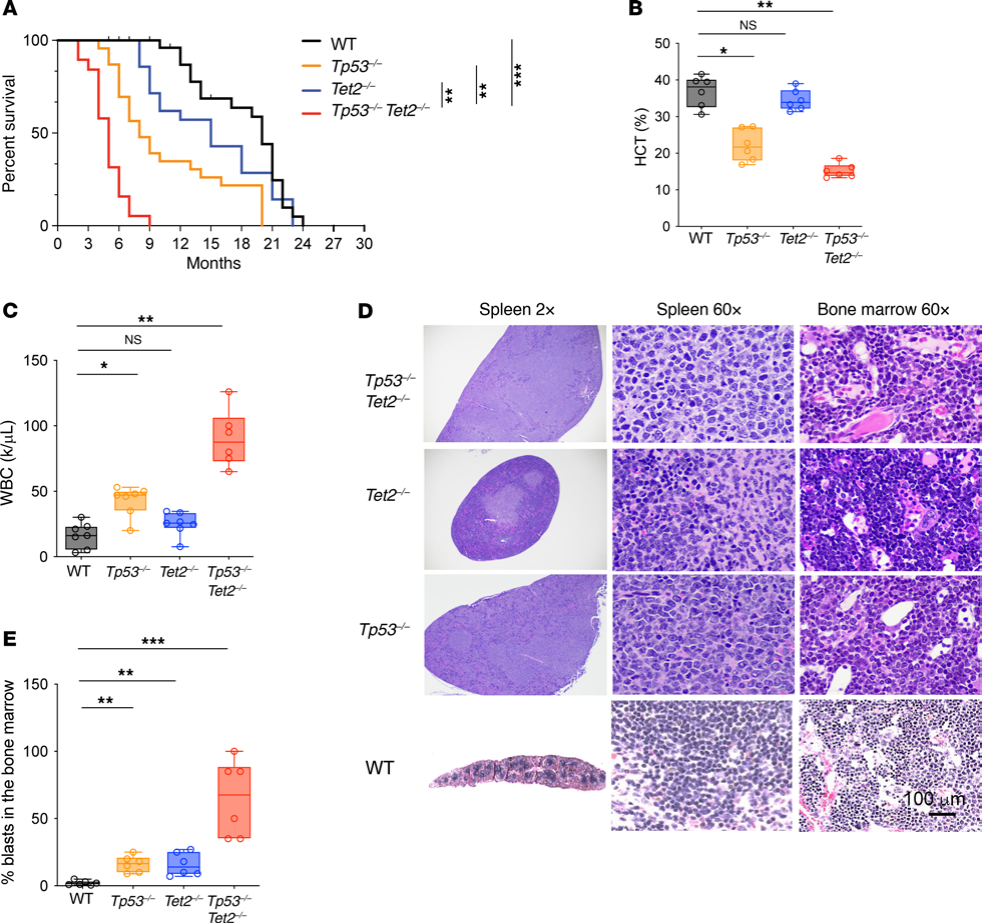
Hematopoietic–cell specific *Tp53*- and *Tet2*-KO mice develop lethal leukemia. (**A**) Kaplan-Meier survival curves of *Tp53^–/–^Tet2^–/–^*, *Tet2^–/–^*, *Tp53^–/–^*, and WT mice. *n* = 32 WT mice, *n* = 23 *Tp53^–/–^* mice, *n* = 19 *Tet2^–/–^* mice, and *n* = 21 *Tp53^–/–^Tet2^–/–^* mice. **P < 0.01; ***P < 0.001. (**B** and **C**) Box-and-whisker plots of (**B**) hematocrit (HCT) and (**C**) white blood cell (WBC) counts of 4-month-old mice with different genotypes. (**D**) H&E staining of spleen and bone marrow in moribund mice of various genotypes. Scale bar: 100 μm; original magnification, ×2 (spleen, left); ×60 (spleen, right, and bone marrow). Data are representative of *n* = 6–7 mice/genotype. (**E**) Box-and-whisker plots of percentage of bone marrow blasts, based on **D** at the age of 4 months. For box-and-whisker plots in **B**, **C**, and **E**, boxes represent median, first, and third quartiles, with whiskers extending to 1.5× interquartile range. *n* = 6–7 mice/genotype. ANOVA with Dunnett’s test was used for significance. *P < 0.05; **P < 0.01; ***P < 0.001. *Tp53^–/–^Tet2*^–/–^, *Vav*-*cre* *Tet2^fl/fl^ Tp53^fl/fl^*; *Tet2^–/–^*, *Vav*-*cre* *Tet2^fl/fl^*; *Tp53^–/–^*, *Vav*-*cre* *Tp53^fl/fl^*; WT, *Vav-cre*.

To study the functional significance of loss of *TET2* in the setting of *TP53* loss-of-function mutations, we generated conditional mice with hematopoietic-specific deletion of both genes by crossing *Vav-cre Tet2^fl/fl^* and *Tp53^fl/fl^* mice. These animals mimicked the clonal combined biallelic mutations in *TP53* and *TET2* seen in patients with AML (Figure 1). *Vav-cre Tet2^fl/fl^* *Tp53^fl/fl^* double-KO mice were viable at birth and born at normal Mendelian ratios, but their median survival (21 weeks) was significantly shorter than that of littermate *Vav-cre* control (WT) or single-gene KO control mice (Figure 2A). In addition, the median survival was shorter than that for previously described *Tet2^–/–^Flt3-ITD* mice (37 weeks) (14). Complete differential blood counts revealed leukocytosis, anemia, and thrombocytopenia in 4-month-old *Vav-cre Tet2^fl/fl^* *Tp53^fl/fl^* mice (Figure 2, B and C). Necropsy of *Vav-cre Tet2^fl/fl^* *Tp53^fl/fl^* mice revealed splenomegaly (Supplemental Figure 1D) and infiltration of immature appearing cells in the liver, lung, lymph node, spleen, and bone marrow (Figure 2, D and E).

**Figure 3:**
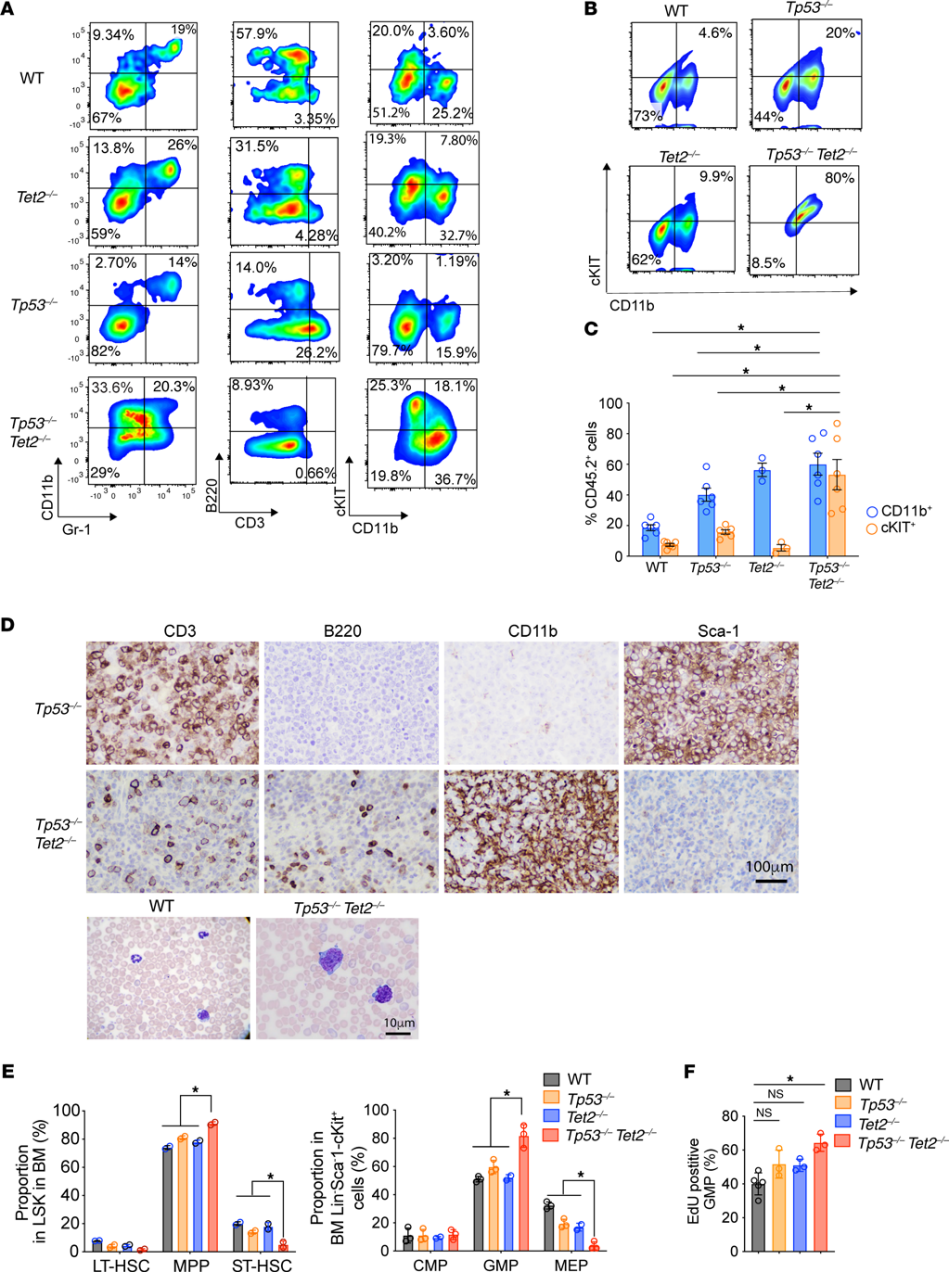
*Tp53/Tet2* double-KO mice develop AML characterized by expansion of granulocyte macrophage progenitors. (**A**) Representative flow cytometry analysis of lineages of CD45^dim^SSC^lo^ cells in spleens from *Tp53^–/–^Tet2^–/–^*, *Tet2*^–/–^, *Tp53^–/–^*, and WT mice at the time of sacrifice (4 months of age). (**B**) Representative flow cytometry analysis of CD11b^+^cKIT^+^ cells in peripheral blood from moribund 4-month-old *Tp53^–/–^Tet2^–/–^* mice and age-matched controls. (**C**) Frequency of CD11b^+^ and cKIT^+^ cells among CD45.2^+^ cells in peripheral blood of moribund 4-month-old *Tp53^–/–^Tet2^–/–^* mice and age-matched controls. *n* = 3–6 mice/group. *P < 0.05. Mean ± SEM. ANOVA with Dunnett’s test was used for significance. (**D**) Top: Immunohistochemical staining of spleens of representative moribund mice for the proteins indicated. Scale bar: 100 μm. Bottom: Wright-Giemsa stain of peripheral blood of *Tp53^–/–^Tet2^–/–^* and littermate WT mice. Data are representative of *n* = 6 mice/group. Scale bar: 10 μm. (**E**) Left: Frequencies of long-term hematopoietic stem cells (LT-HSC), multipotent progenitors (MPP), and short-term HSCs (ST-HSC) among bone marrow lineage-negative Sca1^+^cKIT^+^ (LSK) cells of 16-week-old mice with the indicated genotypes. Right: Frequencies of common myeloid progenitors (CMP), granulocyte macrophage progenitors (GMPs), and megakaryocyte-erythroid progenitors (MEPs) among bone marrow Lin^–^Sca-1^–^cKIT^+^ cells of 16-week-old mice with the indicated genotypes. *n* = 3 mice/group; Mean ± SD. ANOVA with Dunnett’s test. (**F**) Percentage of bone marrow EdU^+^ GMPs in 16-week-old mice with the indicated genotypes. *n* = 5 mice/group. *P<0.05. ANOVA with Dunnett’s test was used for significance. *Tp53^–/–^Tet2^–/–^,* *Vav*-*cre* *Tet2^fl/fl^ Tp53^fl/fl^*; *Tet2^–/–^,* *Vav*-*cre* *Tet2^fl/fl^*; *Tp53^–/–^*, *Vav*-*cre* *Tp53^fl/fl^*; WT, *Vav-cre*.

*Vav-cre Tet2^fl/fl^Tp53^fl/fl^* mice had myeloid lineage profiles of leukemia, with dominant cell populations expressing CD11b and Gr-1, but not a pan–B cell (B220) or a T cell marker (CD3) (Figure 3A). Splenocytes of *Vav-cre Tet2^fl/fl^* *Tp53^fl/fl^* mice that developed AML had substantially increased frequencies of Cd11b^+^Gr-1^–^ and Cd11b^+^Gr-1^+^ myeloid cells as well as increased CD11b^+^cKIT^+^ cells (Figure 3, A and B). Moreover, an abnormally high frequency of cKIT^+^ cells could be detected in peripheral blood of *Vav-cre Tet2^fl/fl^* *Tp53^fl/fl^* mice by 4 months of age (Figure 3C). IHC staining of *Vav-cre Tet2^fl/fl^* *Tp53^fl/fl^* moribund mice that developed AML showed results that were consistent with flow cytometry analysis, revealing myeloid cell expansion and nearly absent lymphoid cells in spleen (Figure 3D). Consistent with previous reports, expressions of CD3 and Sca-1 were increased in *Vav-cre Tp53^fl/fl^* moribund mice developing T acute lymphoblastic leukemia (Figure 3D) (15).

Given the known role of *Tet2* in regulating frequency and self-renewal of hematopoietic stem and progenitor cells (HSPCs) (14), we analyzed bone marrow HSPCs in *Vav-cre Tet2^fl/fl^* *Tp53^fl/fl^* mice and controls at 4 months of age. The frequency of multipotent progenitor cells was significantly increased in *Vav-cre Tet2^fl/fl^* *Tp53^fl/fl^* mice relative to that in other groups, while short-term hematopoietic stem cells (HSCs) were slightly reduced (Figure 3E). We observed an expansion of granulocyte-macrophage progenitors (GMPs), with a marked reduction of megakaryocyte-erythroid progenitors (MEPs) in *Vav-cre Tet2^fl/fl^* *Tp53^fl/fl^* mice compared with that in other groups (Figure 3E). Based on in vivo EdU labeling, GMPs in *Vav-cre Tet2^fl/fl^* *Tp53^fl/fl^* mice with AML were more proliferative than those in controls (Figure 3F). Altogether, we found that concurrent loss of *Tet2* and *Tp53* cooperatively results in development of lethal AML and alters HSPC frequencies.

### Combined deletion of Tp53 and Tet2 enhances HSPC self-renewal.

**Figure 4:**
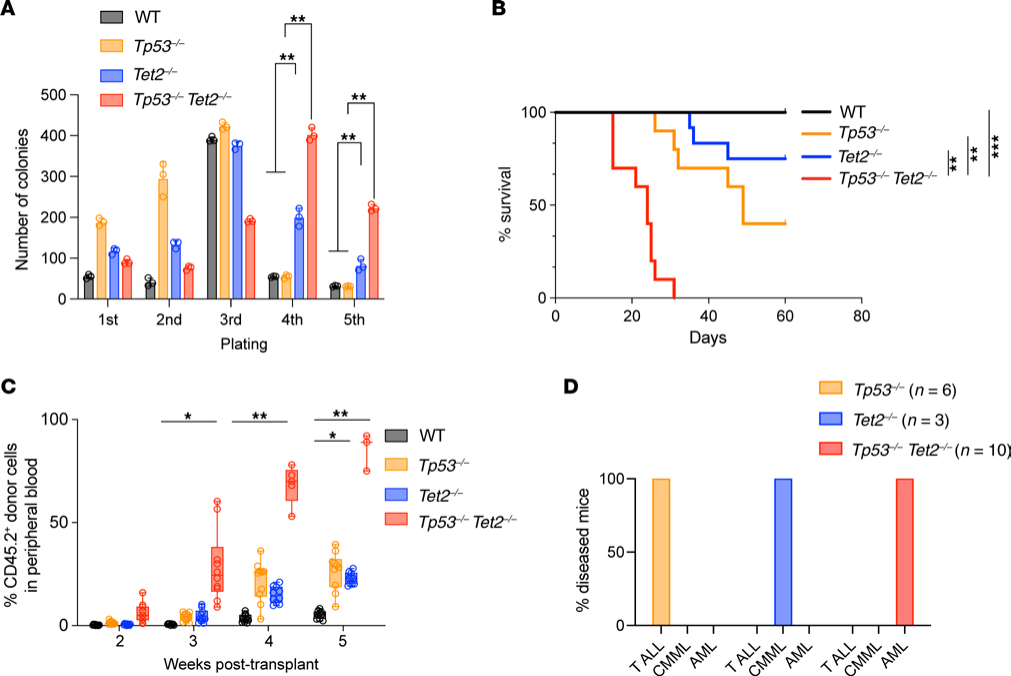
Increased hematopoietic progenitor self-renewal in *Tp53/Tet2* double-KO mice. (**A**) Number of colonies from plating of 10,000 cells from the bone marrow of 16-week-old *Vav*-*cre* *Tp53^fl/fl^Tet2^fl/fl^* mice and controls in methylcellulose. Mean ± SD of 3 technical replicates. (**B**) Kaplan-Meier curves of recipient CD45.1^+^ mice following competitive transplantation of bone marrow cells (1 × 10^6^ cells) from leukemic CD45.2^+^ primary transgenic mice with the indicated genotypes into lethally irradiated recipient mice with CD45.1^+^ supporting bone marrow cells (1 × 10^6^ cells). *n* = 10 WT mice, *n* = 10 *Tp53^–/–^* mice, *n* = 12 *Tet2^–/–^* mice, and *n* = 10 *Tp53^–/–^Tet2^–/–^* mice. (**C**) Box-and-whisker plots of CD45.2^+^ cells in peripheral blood of mice from **B**. Boxes represent median, first, and third quartiles, with whiskers extending to 1.5× interquartile range. *n* = 10 mice/genotype. (**D**) Disease incidence in moribund recipient mice following competitive transplantation of bone marrow cells from primary transgenic mice with the indicated genotypes. T-ALL, T acute lymphoblastic leukemia; CMML, chronic myelomonocytic leukemia; AML, acute myeloid leukemia. *Tp53^–/–^Tet2^–/–^*, *Vav*-*cre* *Tet2^fl/fl^ Tp53^fl/fl^*; *Tet2^–/–^*, *Vav*-*cre* *Tet2^fl/fl^*; *Tp53^–/–^*, *Vav*-*cre* *Tp53^fl/fl^*; WT, *Vav-cre*. A log-rank test was used for survival statistics; otherwise, ANOVA with Dunnett’s test was used for *P* values. *P < 0.05; **P < 0.01; ***P < 0.001.

Deletion of *Tet2* has been repeatedly shown to enhance HSPC self-renewal in vitro and in vivo (16, 17). We therefore next examined the impact of combined *Tet2* and *Tp53* deletion on HSPC self-renewal. Bone marrow cells from *Vav*-*cre Tet2^fl/fl^* *Tp53^fl/fl^* mice sustained up to 5 rounds of plating, at which time they yielded larger numbers of cells in culture relative to controls (Figure 4A). To evaluate self-renewal in vivo, we conducted competitive transplantation by transplanting 1 × 10^6^ bone marrow cells from CD45.2^+^ *Vav*-*cre Tet2^fl/fl^* *Tp53^fl/fl^*, *Vav*-*cre Tet2^fl/fl^,* *Vav*-*cre Tp53^fl/fl^*, or *Vav*-*cre* control mice together with congenic CD45.1^+^ competitor cells at 1:1 ratio into lethally irradiated CD45.1^+^ recipient mice (Figure 4B). At these cell doses, recipients of *Vav*-*cre Tet2^fl/fl^* *Tp53^fl/fl^* cells developed lethal myeloid neoplasms and died by 33 days after transplantation (Figure 4B). Robust engraftment was observed, with 30%–60% of CD45.2^+^ donor cells readily detectable in peripheral blood of recipients of *Vav*-*cre Tet2^fl/fl^* *Tp53^fl/fl^* bone marrow cells as early as 3 weeks after engraftment (Figure 4C). Unlike mice engrafted with *Vav-cre* *Tet2^fl/fl^* *Tp53^fl/fl^* cells, mice receiving *Vav-cre Tet2^fl/fl^* or *Vav-cre Tp53^fl/fl^* donor cells had longer survival times and died from a chronic myelomonocytic leukemia–like disease (for *Vav*-*cre Tet2^fl/fl^* mice) or T cell malignancies (for *Vav-cre Tp53^fl/fl^* mice) (Figure 4D).

### TLR2/A20/noncanonical NF-κB pathway–mediated proinflammatory signaling promotes myeloid leukemia development in TET2/TP53-mutant progenitors.

**Figure 5:**
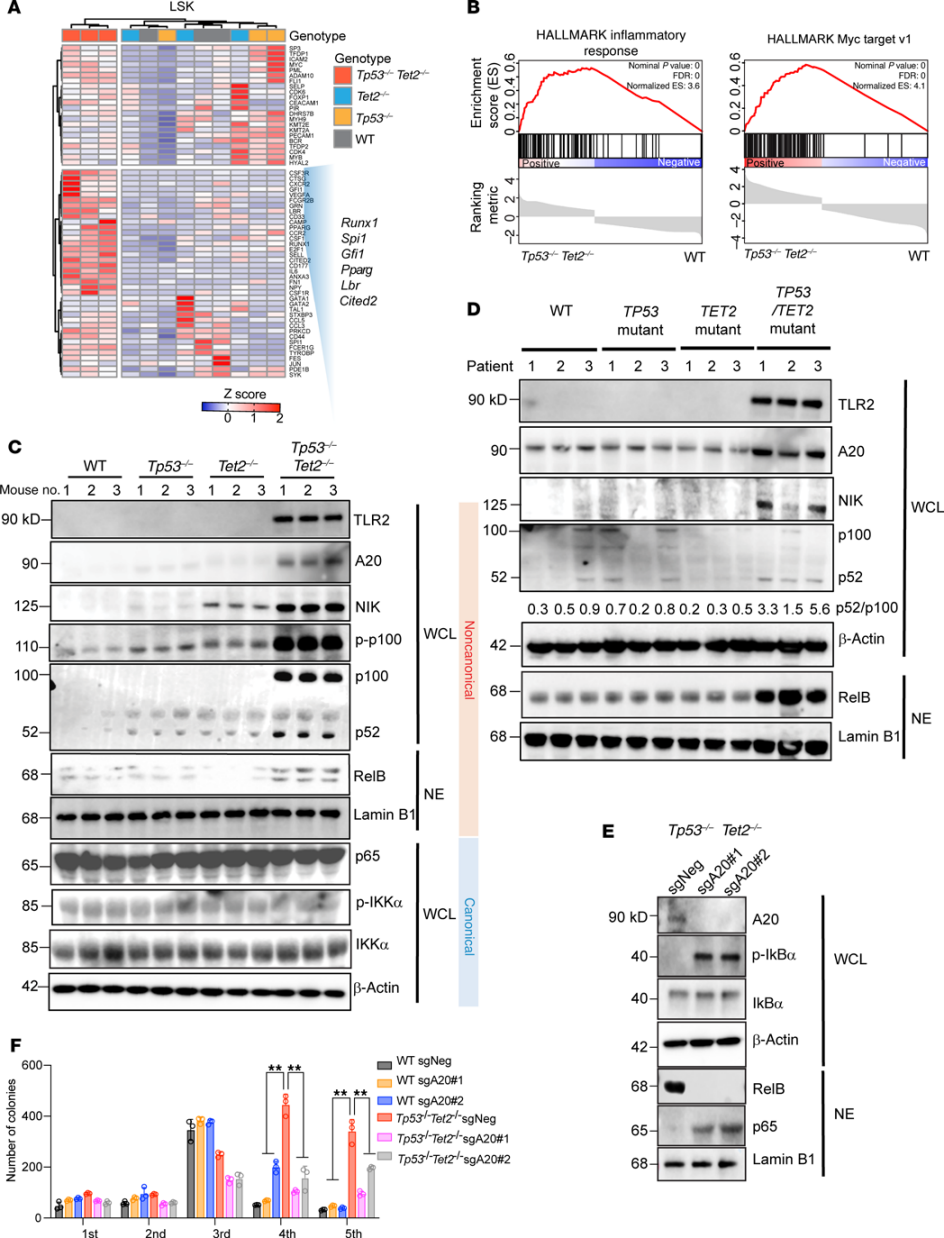
Myeloid predisposition and enhanced innate immune signaling in *Tp53^–/–^Tet2^–/–^* precursor cells. (**A**) Heatmap of top differentially expressed genes (FDR < 0.05) within the myeloid differentiation pathway of LSK cells from 4-month-old mice with different genotypes. Key myeloid transcriptional factors upregulated in *Tp53/Tet2* double-KO LSK cells relative to other groups are highlighted. (**B**) GSEA plots of inflammatory responses and the Myc pathway in LSK cells from *Tp53^–/–^Tet2^–/–^* mice with AML relative to control mice. (**C**) Western blot of TLR2, A20, noncanonical NF-κB pathway components (NIK, p52/p100, and phosphorylated p100 [p-p100] in whole-cell lysate [WCL] and RelB in nuclear extract [NE]) and canonical NF-κB pathway members (p65, phosphorylated-IKKα [p-IKKα], and IKKα) in cKit^+^ bone marrow cells from age-matched mice with indicated genotypes (3 mice per group). β-Actin and Lamin B1 served as housekeeping controls in WCL and NE, respectively. (**D**) Western blot of TLR2, A20, NIK, and p52/p100 in WCL and RelB in NE from patients with combined *TET2* and *TP53* mutations, either mutation alone, or neither *TET2* or *TP53* mutations (WT). β-Actin and Lamin B1 served as housekeeping controls in WCL and NE, respectively. Analysis of fold change normalized to the control lane is shown below immunoblot where indicated. (**E**) Western blot of A20, phosphorylated IkBα (p-IkBα), and total IkBα in WCL and RelB and RelA in NE in cKit^+^ bone marrow cells of *Tp53^–/–^Tet2^–/–^* mice treated with control (sgNeg) or 1 of 2 A20-targeting sgRNAs. β-Actin and Lamin B1 served as housekeeping controls in WCL and NE, respectively. (**F**) Mean number of methylcellulose colonies in cells from **E** and WT bone marrow cells treated with control or 1 of 2 A20-targeting sgRNAs. Mean ± SD shown. **P < 0.01. Results are representative of 2–3 independent experiments. *Tp53^–/–^Tet2^–/–^*, *Vav*-*cre* *Tet2^fl/fl^ Tp53^fl/fl^*; *Tet2^–/–^*, *Vav*-*cre* *Tet2^fl/fl^*; *Tp53^–/–^*, *Vav*-*cre* *Tp53^fl/fl^*; WT, *Vav-cre*.

To understand the molecular mechanisms underlying AML transformation upon combined mutation in *TET2* and *TP53*, we performed bulk RNA-Seq of Lin^–^cKIT^+^ myeloid progenitors (LK) and Lin^–^cKIT^+^Sca-1^+^ (LSK) cells from diseased *Vav*-*cre Tet2^fl/fl^* *Tp53^fl/fl^* mice developing AML and age-matched *Vav*-*cre Tp53^fl/fl^*, *Vav-cre Tet2^fl/fl^*, and WT mice. The myeloid transcriptional factors *RUNX1*, *SPI1*, *GFI1*, *PPARG,* *LBR*, and *CITED2* were upregulated in expression in *Vav*-*cre Tet2^fl/fl^* *Tp53^fl/fl^* LSK cells compared with other groups of cells (Figure 5A, Supplemental Figure 2A, and Supplemental Table 1), and gene set enrichment analysis (GSEA) revealed an enrichment in gene signatures in LSK cells related to myeloid differentiation and AML transformation (such as immortalized *HOXA9*, *MEIS1_*up, *GATA2* targets-up, and myeloid cell development up) (Supplemental Figure 2B). Additionally, gene signatures associated with Myc and inflammatory responses were enriched in *Vav*-*cre Tet2^fl/fl^* *Tp53^fl/fl^* AML versus WT LSK cells (Figure 5B). In contrast, T lymphocyte commitment gene sets were enriched in *Vav-cre Tp53^fl/fl^* LSK progenitors, consistent with findings that *Tp53* loss alone in *Vav*-*cre Tp53^fl/fl^* mice primarily drove T acute lymphoblastic leukemia (Supplemental Figure 2, C and D).

Beyond genes involved in myeloid cell commitment and oncogenes, we observed increased expression of *Tnfaip3* and *Tlr2* in *Vav*-*cre Tet2^fl/fl^* LK cells, compared with WT LK cells, which was further increased in *Vav*-*cre Tet2^fl/fl^* *Tp53^fl/fl^* LK cells (Supplemental Figure 2E). *Tnfaip3* encodes A20 deubiquitinase, and *Tlr2* encodes TLR2. A20 can mediate HSC transformation by activating noncanonical NF-κB signaling (17). Inhibition of A20 expression can prevent TLR-TRAF6–primed *TET2-*mutant MDS from progressing to leukemia (18). Thus, we evaluated A20 and associated canonical/noncanonical NF-κB pathway component protein levels. Our data showed increased levels of TLR2, A20, and noncanonical NF-κB pathway components, including NIK, p100, p52, phosphorylated p100, and elevated nuclear-localized RelB, in *Vav*-*cre Tet2^fl/fl^* *Tp53^fl/fl^* mice in comparison to *Vav*-*cre Tet2^fl/fl^*, *Vav*-*cre Tp53^fl/fl^* or WT mice (Figure 5C). In contrast, canonical NF-κB pathway members p65, phosphorylated IKKα, and IKKα were not upregulated in *Vav*-*cre Tet2^fl/fl^* *Tp53^fl/fl^* mice (Figure 5C). Similarly, TLR2, A20, NIK and the ratio of p52/p100 were consistently elevated in bone marrow or blood cells from patients with AML harboring comutations in *TET2* and *TP53* (Figure 5D and Supplemental Table 2). In addition, we observed more nuclear-localized RelB in cells from patients with *TP53/TET2* comutations compared with those with single mutations (Figure 5D).

Previous studies have suggested that *TET2-*deficient HSPCs express A20 and exhibit noncanonical NF-κB activation. Additionally, LPS can enhance the expansion of *Tet2*-KO HSCs by activating NF-κB signaling (18, 19). Furthermore, LPS promotes malignant transformation in *Tet2*-mutant mice by accelerating the production of MHC II^hi^ monocytes (20). Infection-derived LPS drives preleukemic myeloproliferation in these mice, a process that can be reversed by antibiotic treatment (19). To assess TLR function in leukemia cells, we measured in vitro responses of mouse cells to the TLR2 agonist, PAM3CSK4. Upon PAM3CSK4 stimulation, the levels of A20, NIK, and phosphorylated p100 and the ratio of p52/p100 were elevated in cells derived from WT and single-mutant mice, as expected (Supplemental Figure 2F). However, the extent of upregulation of these proteins was substantially higher in *Vav-cre Tet2^fl/fl^* *Tp53^fl/fl^* cells compared with WT or single-mutant cells. These findings indicate that the noncanonical NF-kB signaling pathway was hyperactivated in response to TLR2 stimulation in *Vav*-*cre Tet2^fl/fl^* *Tp53^fl/fl^* murine hematopoietic cells (Supplemental Figure 2F). In contrast, canonical NF-κB pathway components, including p50, IkBα, and phosphorylated IkBα, did not exhibit differential changes in response to TLR2-induced activation in *Vav*-*cre Tet2^fl/fl^* *Tp53^fl/fl^* cells (Supplemental Figure 2F).

The data above suggest that combined loss of *TET2* and *TP53* enhances sensitivity to innate immune signaling, revealing cooperative upregulation of myeloid transcription factor expression and enhancement of proinflammatory signaling. We next sought to determine the functional requirement for A20 in the maintenance of *Tp53/Tet2* double-mutant leukemia. We genetically depleted A20 in mouse cKit^+^ bone marrow cells from *Vav*-*cre Tet2^fl/fl^* *Tp53^fl/fl^* mice (Figure 5E). A20 deletion was confirmed by Western blot. Deletion of A20 decreased the level of nuclear RelB while increasing nuclear localization of p65, and promoted phosphorylation of IkBα (Figure 5E). Additionally, A20 loss strikingly reduced the serial replating capacity of *Tp53/Tet2* double-KO murine AML cells in vitro (Figure 5F).

### Tumor-intrinsic transcriptional features are associated with distinct leukemia phenotypes.

**Figure 6:**
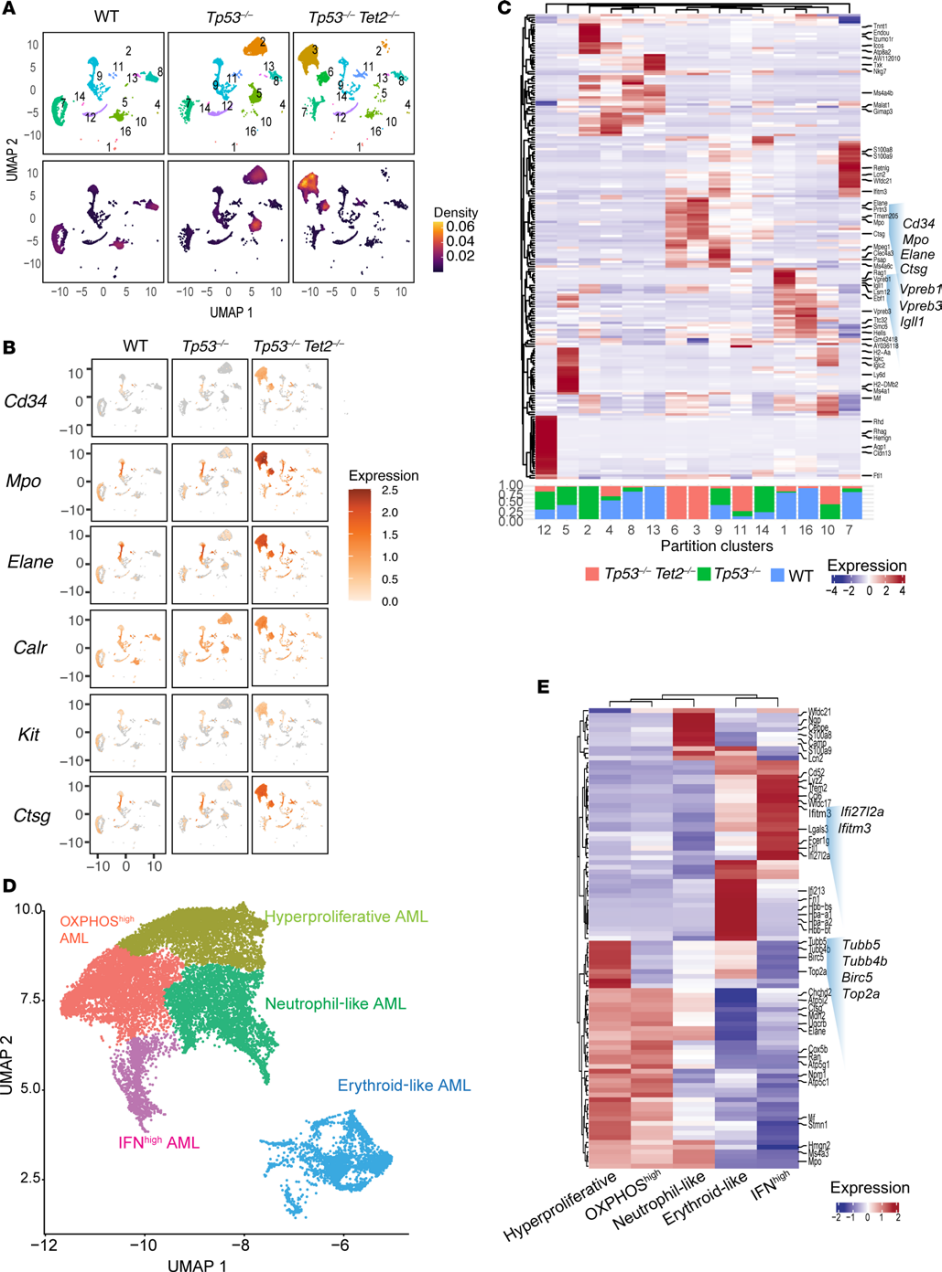
*Tp53/Tet2* comutant AML displays unique transcriptional signatures. (**A**) UMAP plots of single-cell transcriptomes of bone marrow mononuclear cells from *Vav*-*cre* control mice (WT), *Vav*-*cre* *Tp53^fl/fl^* mice, or *Vav*-*cre* *Tet2^fl/fl^ Tp53^fl/fl^* mice. Cell density (2D kernel density estimate mapped to color scale) plots are shown underneath. *n* = 2 mice/group; 3 weeks after engraftment into CD45.1^+^ mice. (**B**) UMAP projection of the expression of selected myeloid marker genes. Expression is displayed by color scale as log_10_-transformed expression (size factor normalized unique molecular identifier counts). (**C**) Heatmap with hierarchical clustering showing top differentially expressed genes in clusters from **A** across the different groups of mice. The normalized proportion allocations of cells of various phenotypes in each cluster are shown as stacked bar plots underneath the heatmap. (**D**) Annotated subpopulations (Leiden clusters) of AML blast clusters (partition clusters 3 and 6) (49) showing identified AML subtypes. (**E**) Heatmap showing top differentially expressed genes identified in each AML subtype from **D**. *Tp53^–/–^Tet2^–/–^*, *Vav*-*cre* *Tet2^fl/fl^ Tp53^fl/fl^*;*Tp53^–/–^*, *Vav*-*cre* *Tp53^fl/fl^*; WT, *Vav-cre*.

Given the heterogeneity of malignant cells in leukemia, we next performed single-cell RNA-Seq (scRNA-Seq) of bone marrow cells from *Vav*-*cre Tet2^fl/fl^* *Tp53^fl/fl^* mice as well as WT and *Vav*-*cre* *Tp53^fl/fl^* mice. Uniform manifold approximation and projection (UMAP) dimension reduction of all samples yielded 16 distinct partition clusters (Figure 6A). Cells in clusters 3 and 6, predominantly contributed by *Vav*-*cre Tet2^fl/fl^* *Tp53^fl/fl^* AML cells, showed enrichment of myeloid leukemia marker genes as well as myeloid-associated genes (Figure 6, B and C) (21). To further evaluate intratumoral heterogeneity in *TP53/TET2* comutant AML, we further fragmented AML clusters 3 and 6 and identified 5 distinct clusters of AML cells, including OXPHOS^hi^, IFN^hi^, hyperproliferative, neutrophil-like, and erythroid-like AML populations (Figure 6, D and E) (22).The IFN^hi^ AML population displayed enrichment in myeloid suppressor cell markers, macrophage myeloid differentiation markers (*Ccl6*), the eosinophil IgE receptor (*Fcer1g*), and *Cd52,* which is highly expressed on leukemia progenitor cells (Figure 6E). Endosome-associated factor, *Ifitm3*, showed aberrantly high expression in the IFN^hi^ cells (Figure 6E). Genes associated with secondary granules were upregulated in neutrophil-like AML (23). The hyperproliferative AML subcluster displayed features of G_1_/S phase transition with histone gene expression and mitotic drivers. OXPHOS^hi^ AML cells express a variety of mitochondrial respiratory chain–related genes (24). Erythroid-like AML (cluster 6) is characterized by the expression of hemoglobin genes alongside myeloid markers such as *Elane*, *Mpo*, and *Cd34*. However, this erythroid-like cluster differs from acute erythroid/megakaryocytic leukemia, which often originates from the common MEP. In *Vav*-*cre* *Tet2^fl/fl^ Tp53^fl/fl^* mice, MEPs were notably reduced, correlating with the anemia and thrombocytopenia observed in these mice (Figure 2B). Clusters 3 and 6, as well as the associated 5 subclusters were negative for lineage genes but coexpressed HSC/multipotent progenitor cell markers *Cd48*, *Cd150*, *Kit*, and *Sca1* (Supplemental Figure 3, A and B).

To evaluate the similarity between mouse and human AML, we performed cellular indexing of transcriptome and epitope sequencing (CITE-Seq) with 12 patients with *TP53/TET2*-mutant AML, 6 patients with *TP53-*mutant AML, 6 patients with *TET2*-mutant AML, and 6 patients with WT AML and projected mouse scRNA-Seq data onto human AML CITE-Seq data (Supplemental Figure 3, C and D). This comparative analysis revealed that *Vav-cre Tet2^fl/fl^* *Tp53^fl/fl^* murine AML cells significantly align transcriptionally with *TP53/TET2* comutant human AML cells compared with single-mutant or WT control cells (binomial test, *P* < 2.2 × 10^–16^, predicted probability = 0.25, observed probability = 0.78, 95% confidential interval = 0.76) (Supplemental Figure 3, E and F). This suggests that distinct clusters 3 and 6 of *Vav*-*cre Tet2^fl/fl^* *Tp53^fl/fl^* murine AML closely mirror the cellular heterogeneity and transcriptional signatures observed specifically in the corresponding *TP53/TET2* comutant human AML.

Cluster 1, which was enriched in wild-type, single-KO, and double-KO cells, showed high expression of B cell lineage marker genes, *Cd19*, *Cd24a*, *Pax5*, and *Cd79a*, and genes with functions in class-switch recombination and somatic hypermutation (Figure 6C and Supplemental Figure 4A). In contrast, T cell markers were expressed in cluster 2, which is specific for *Vav*-*cre Tp53^fl/fl^* cells (Supplemental Figure 4B).

Next, we evaluated molecular mechanisms that may drive different leukemia fates upon combined *TET2* and *TP53* deletion. *Vav*-*cre Tet2^fl/fl^* *Tp53^fl/fl^* AML bone marrow cells formed a distinct cluster compared with *Vav*-*cre Tp53^fl/fl^* and WT mouse–derived cells (Louvain cluster 19; Supplemental Figure 5, A and B). The cells in cluster 19 were predominantly from clusters 3 and 6 (Figure 6A). The expression levels of *Klf4* and *Itga4* were progressively elevated during transition from early precursors to AML, peaking in cluster 19 (Supplemental Figure 5C).

### GMPs in Tp53 and Tet2 double-KO AML exhibit distinct transcriptional signatures and acquire leukemia-initiating capacity.

**Figure 7:**
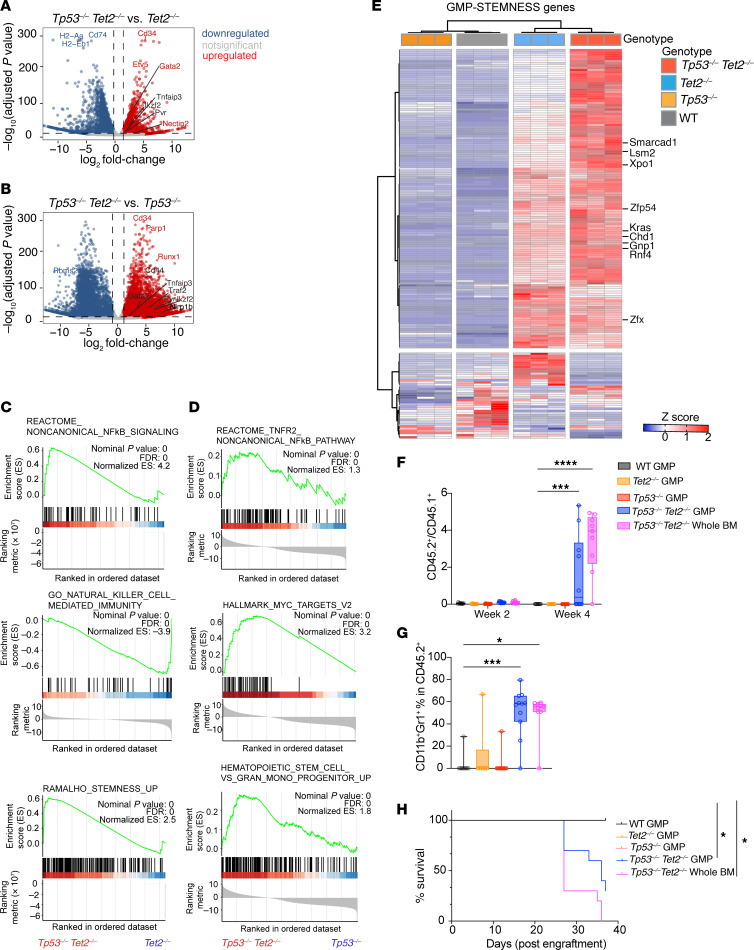
Mutant *Tp53* and *Tet2* cooperatively transform murine GMP progenitors. Volcano plots depicting top differentially expressed genes in GMPs (Lin^–^cKIT^+^Sca1^–^ CD16/32^+^ CD34^+^) from (**A**) *Tp53^–/–^Tet2^–/–^* versus *Tet2^–/–^* or (**B**) *Tp53^–/–^Tet2^–/–^* versus *Tp53^–/–^* mice. (**C**) GSEA plots of pathways enriched in GMPs from *Tp53^–/–^Tet2^–/–^* mice with AML relative to *Tet2^–/–^* control mice. (**D**) As in **C** but for GMPs from *Tp53^–/–^Tet2^–/–^* mice with AML relative to *Tp53^–/–^* control mice. (**E**) Heatmap of top differentially expressed genes (adjusted *P* < 0.05) within the STEMNESS_UP pathway of GMP cells from 4-month-old mice with different genotypes. Key genes upregulated in *Tp53^–/–^Tet2^–/–^* GMP cells are highlighted. (**F**) CD45.2^+^ (mutant) to CD45.1^+^ (WT) cell ratios and (**G**) CD11b^+^Gr1^+^ percentage in CD45.2^+^ cells in the peripheral blood of recipient mice engrafted with CD45.2^+^ cell types (either GMPs or whole bone marrow [BM]) from the animals with the indicated genotypes. For box-and-whisker plots, boxes represent median, first, and third quartiles, with whiskers extending to 1.5× interquartile range. *P* values are shown (ANOVA with Dunnett’s test). ***P < 0.001; **** *P* < 0.0001. (**H**) Kaplan-Meier curves of lethally irradiated CD45.1^+^ mice receiving whole BM or GMP cells from mice with different genotypes. Log-rank test was used for survival statistics. *P < 0.05. *Tp53^–/–^Tet2^–/–^*, *Vav*-*cre* *Tet2^fl/fl^ Tp53^fl/fl^*; *Tet2^–/–^*, *Vav*-*cre* *Tet2^fl/fl^*; *Tp53^–/–^*, *Vav*-*cre* *Tp53^fl/fl^*; WT, *Vav-cre*.

Given the expansion of GMPs in *Vav-cre Tet2^fl/fl^* *Tp53^fl/fl^* mice, we also sought to evaluate gene expression specifically in *Vav-cre Tet2^fl/fl^* *Tp53^fl/fl^* GMPs relative to controls. We therefore sorted GMPs from *Vav-cre Tet2^fl/fl^* *Tp53^fl/fl^* mice or control mice and performed RNA-Seq. Gene expression of GMPs from *Vav-cre Tet2^fl/fl^* *Tp53^fl/fl^* mice was profoundly altered in comparison to that in single-mutant controls. Compared with *Vav-cre Tet2^fl/fl^* GMPs, MHC class II genes, *H2-Aa* and *H2-Eb1,* and MHC II trafficking adaptor gene, *Cd74*, were significantly downregulated in *Vav-cre Tet2^fl/fl^* *Tp53^fl/fl^* GMPs (Figure 7A and Supplemental Table 3). In sharp contrast, stem cell gene, *Cd34*; lineage gene, *Gata2*; and leukemia stem cell regulator, *Ikzf2*; as well as *Tnfaip3*, *Pvr* (*Cd155*), and *Nectin2* (*Cd112*), were upregulated in *Vav-cre Tet2^fl/fl^* *Tp53^fl/fl^* GMPs (Figure 7A). In comparison to *Vav-cre Tp53^fl/fl^* GMPs, tumor suppressor/p21 stabilizer, *Rbms2*, was downregulated, while transcripts of stem cell genes, *Cd34* and *Cd44*; leukemia enhancer genes, *Runx1*, *Ikzf2*, *and Gata2*; inflammasome gene, *Nlrp1b*; and *Tnfaip3*, *Pvr* (*Cd155*), and *Traf2*, were significantly upregulated in GMPs from *Vav-cre Tet2^fl/fl^* *Tp53^fl/fl^* mice (Figure 7B and Supplemental Table 4). Moreover, gene expression signatures of noncanonical NF-κB signaling, MYC targets, and genes enriched in normal HSCs versus GMPs were positively enriched in *Vav-cre Tet2^fl/fl^* *Tp53^fl/fl^* GMPs compared with those from single-KO GMPs (Figure 7, C and D, and Supplemental Tables 5 and 6). Conversely, genes associated with NK cell–mediated immunity were negatively enriched in *Vav-cre Tet2^fl/fl^* *Tp53^fl/fl^* GMPs. These observations are consistent with our findings in LK cells (Figure 5B).

*Tp53* loss and *Tet2* depletion cooperatively upregulated transcriptional factors regulating stemness (25), including *Mdfic*, *Zfp54*, *Zfx*, and *Rnf4*; chromatin-remodeling helicases of the SNF2/SWI2 family, including *Chd1* and *Smarcad1*; and other well-known regulators of leukemogenesis, such as *Lsm1*, *Xpo1*, *Kras*, and *Gnb1*, in GMP progenitors (Figure 7E and Supplemental Table 7). Furthermore, *Myc* was elevated in GMPs in *Vav-cre Tet2^fl/fl^* *Tp53^fl/fl^* mice (Supplemental Figure 6A).

Compared with WT GMPs, *Vav-cre* *Tet2^fl/fl^* *Tp53^fl/fl^* GMPs exhibited a greater number of differentially expressed genes than the single-KO controls (Supplemental Figure 6B). Of these differentially expressed genes, 1,054 were upregulated and 1,616 were downregulated, with these changes being unique to the *Vav*-*cre Tet2^fl/fl^* *Tp53^fl/fl^* GMPs. This suggests a cooperative effect between *Tp53* depletion and *Tet2* loss.

The enrichment of stemness gene signature in GMPs from *Vav*-*cre* *Tet2^fl/fl^* *Tp53^fl/fl^* mice suggested that these cells may acquire self-renewal and leukemia-initiating cell features. To test this hypothesis, we sorted CD45.2^+^ GMPs from WT, *Vav-cre* *Tet2^fl/fl^*, *Vav*-*cre Tp53^fl/fl^*, and *Vav*-*cre Tet2^fl/fl^* *Tp53^fl/fl^* mice and engrafted them into lethally irradiated recipient mice with CD45.1^+^ supporting bone marrow mononuclear cells. We also included a positive control cohort of CD45.1^+^ mice engrafted with whole bone marrow mononuclear cells from *Vav-cre Tet2^fl/fl^ Tp53^fl/fl^* mice. Four weeks after transplant, higher percentages of myeloid CD45.2^+^ cells were found in mice engrafted with *Vav-cre Tet2^fl/fl^ Tp53^fl/fl^* GMPs or whole bone marrow compared with other groups (Figure 7, F and G). Moreover, transplantation of GMPs from *Vav-cre Tet2^fl/fl^ Tp53^fl/fl^* mice resulted in death of recipient animals at a rate comparable to that of recipient mice engrafted with unfractionated bone marrow mononuclear cells (Figure 7H). These data indicated that *Vav*-*cre Tet2^fl/fl^* *Tp53^fl/fl^* GMP cells have leukemia-initiating capacity.

### TP53 and TET2 double-mutant AML is characterized by T/NK cell exhaustion and monocytic MDSC-like cell expansion.

**Figure 8:**
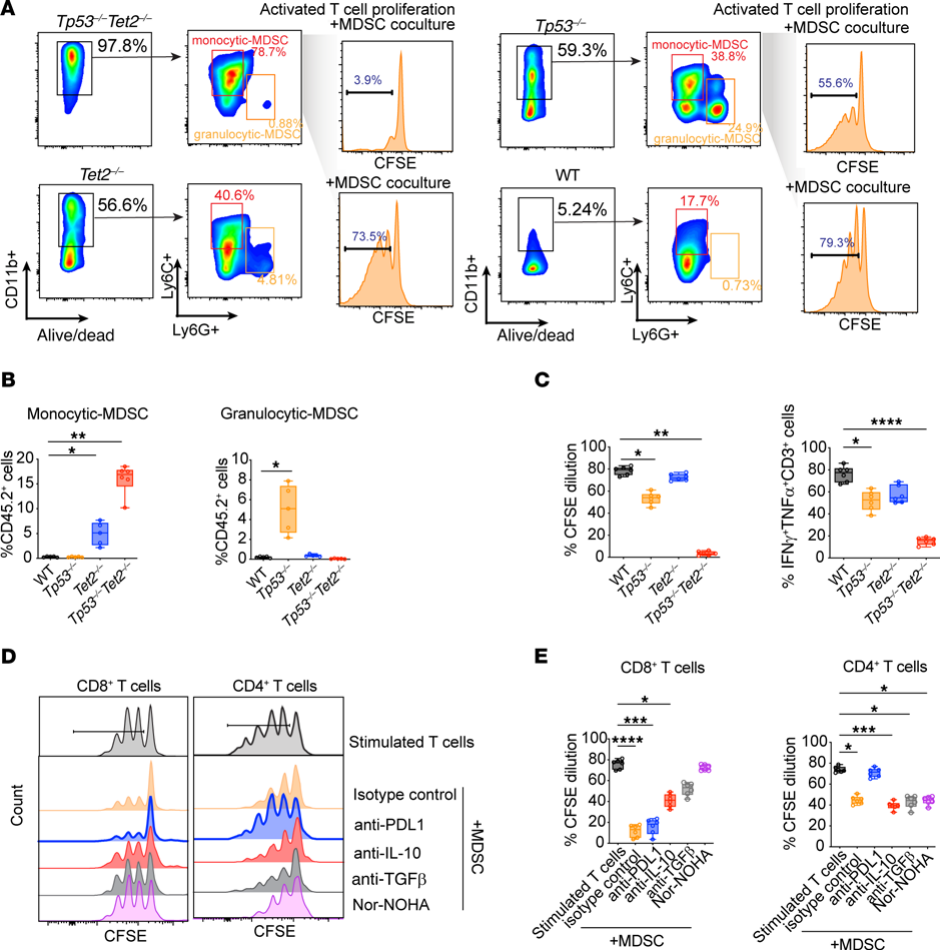
Monocytic MDSCs emerge in *Tp53^–/–^Tet2^–/–^* leukemic environment to cause T cell dysfunction. (**A**) Representative FACS plots of the frequency of monocytic (CD11b^+^Ly6G^–^Ly6C^+^) and granulocytic MDSCs (CD11b^+^Ly6G^+^Ly6C^–^) in splenocytes from *Vav*-*cre* *Tet2^fl/fl^Tp53^fl/fl^* (*Tp53^–/–^Tet2^–/–^*), *Vav*-*cre* *Tet2^fl/fl^* (*Tet2^–/–^),* *Vav*-*cre* *Tp53^fl/fl^* (*Tp53^–/–^*), and WT mice (*n* = 6 mice/group). Suppression of T cell proliferation by isolated MDSCs: T cells from WT mouse spleens were isolated and stimulated with anti-CD3/CD28 Dynabeads. Subsequently, T cells were cocultured with MDSCs isolated from *Tp53^–/–^Tet2^–/–^*, *Tet2^–/–^,* *Tp53^–/–^*, and WT mouse spleens for 72 hours. Cell division was measured with CFSE proliferation assays. *n* = 6 mice/group. (**B**) Quantification of the percentage of monocytic and granulocytic MDSCs in CD45.2^+^ splenocytes across genotypes, as in **A**. (**C**) Quantification of the percentage of CFSE dilution in T cell proliferation and percentage of cytokine-expressing T cells upon coculture with MDSCs isolated from *Tp53^–/–^Tet2^–/–^*, *Tet2^–/–^,* *Tp53^–/–^*, and WT mice. *P* values are shown (ANOVA with Dunnett’s test). *P < 0.05; **P < 0.01; ****P < 0.0001. (**D**) Representative flow plot showing the division of CD4^+^ or CD8^+^ T cells with CFSE staining in response to MDSC coculture in the presence or absence of blocking antibodies or arginase inhibitor (Nor-NOHA). cKit^+^CD11b^+^Ly6C^+^Ly6G^–^ monocytic MDSCs from *Tet2^–/–^Tp53^–/–^* mice were sorted and cocultured with anti-CD3/CD28–activated congenic CD8^+^ or CD4^+^ T cells at 1:8 ratio in the presence of IL-2 for 72 hours. In the selected conditions, coculture was performed in the presence of blocking antibodies or Nor-NOHA (N-Hydroxy-nor-L-arginine). (**E**) Percentage of CFSE dilution in T cell proliferation in T cell–MDSC coculture under different treatment conditions. For box-and-whisker plots, boxes represent median, first, and third quartiles, with whiskers extending to 1.5 × interquartile range. *n* = 6 mice/group. *P* values are shown (ANOVA with Dunnett’s test). *P < 0.05; **P < 0.01; ***P < 0.001; ****P < 0.0001. *Tp53^–/–^Tet2^–/–^*, *Vav*-*cre* *Tet2^fl/fl^ Tp53^fl/fl^*; *Tet2^–/–^*, *Vav*-*cre* *Tet2^fl/fl^*; *Tp53^–/–^*, *Vav*-*cre* *Tp53^fl/fl^*; WT, *Vav-cre*.

One recent study proposed that mutant *TP53* creates an immunosuppressive bone marrow environment in patients with MDS (26). We found that concomitant loss of *Tp53* and *Tet2* promotes expansion of CD11b^+^Ly6C^+^Ly6G^–^ monocytic myeloid-derived suppressor cell–like (MDSC-like) cells but not CD11b^+^Ly6C^–^Ly6G^+^ granulocytic MDSC-like cells (Figure 8, A and B). When isolated and cocultured with T cells isolated from spleens of WT mice, monocytic MDSCs suppressed T cell proliferation in vitro (Figure 8, B and C). In addition, IFN-γ and TNF-α production was reduced in T cells cocultured with MDSCs derived from *Vav-cre* *Tet2^fl/fl^* *Tp53^fl/fl^* AML. In contrast, MDSCs from WT or *Vav-cre* *Tet2^fl/fl^* or *Vav-cre* *Tp53^fl/fl^* mice had less of an impact on T cell activation (Figure 8C).

To understand whether the suppressive roles of monocytic MDSCs are mediated via soluble factors or direct receptor-ligand engagement, the MDSC and T cell coculture experiments above were repeated in the presence or absence of neutralizing antibodies against IL-10, TGF-β, and PD-L1 or Nor-NOHA (N-Hydroxy-nor-L-arginine) to inhibit arginase in MDSCs. CD4^+^ or CD8^+^ T cells were stained with CFSE, and the impact of MDSC coculture in the presence or absence of neutralizing antibodies or Nor-NOHA was evaluated. This assay clearly revealed that monocytic MDSCs suppressed the proliferation of CD4^+^ or CD8^+^ T cells (Figure 8, D and E). Importantly, however, the monocytic MDSC-mediated inhibition of CD8^+^ T cell proliferation and activation (indicated by elevated IFN-γ and TNF-α production) was rescued fully by Nor-NOHA (and only partially by anti–IL-10 or anti–TGF-β antibodies) (Figure 8E and Supplemental Figure 7A). Monocytic MDSC-mediated inhibition of CD4^+^ T cell proliferation and activation was rescued by anti–PD-L1 antibodies (Figure 8E and Supplemental Figure 7A). These data suggest that monocytic MDSC-mediated CD8^+^ T cell dysfunction is mediated via a soluble factor (likely via L-arginine deprivation), while the suppressive effect on CD4^+^ T cells is mediated via direct PD1–PD-L1 engagement. In agreement with in vitro assays, there were more TIGIT^+^LAG3^+^CTLA4^+^ dysfunctional T cells but fewer TIGIT^–^LAG3^–^CTLA4^–^ CD3^+^ normal T cells in *TP53*/*TET2* comutant patient samples (Supplemental Figure 7, B and C). To assess the presence of monocytic MDSCs in humans, we performed spectral flow cytometry utilizing markers, such as CD66b, CD16, CD33, CD11b, CD14, and CD15. Our findings indicate that, while granulocytic MDSCs were elevated in patients harboring *TP53* mutations, monocytic MDSCs were significantly increased in individuals with concurrent *TP53* and *TET2* mutations (Supplemental Figure 8, A–C). In agreement with spectral flow analysis, CITE-Seq analysis uncovered that, at the single-cell level, the frequency of monocytic MDSCs (CD33^+^CD11b^+^HLA-DR^lo/–^CD14^+^CD15^–^ cells) was drastically elevated in the microenvironment of AML with *TP53* and *TET2* comutations (Supplemental Figure 8, D–H). This suggests that an increased abundance of monocytic MDSCs is a characteristic feature in both murine and human AML harboring *TP53* and *TET2* comutations.

**Figure 9:**
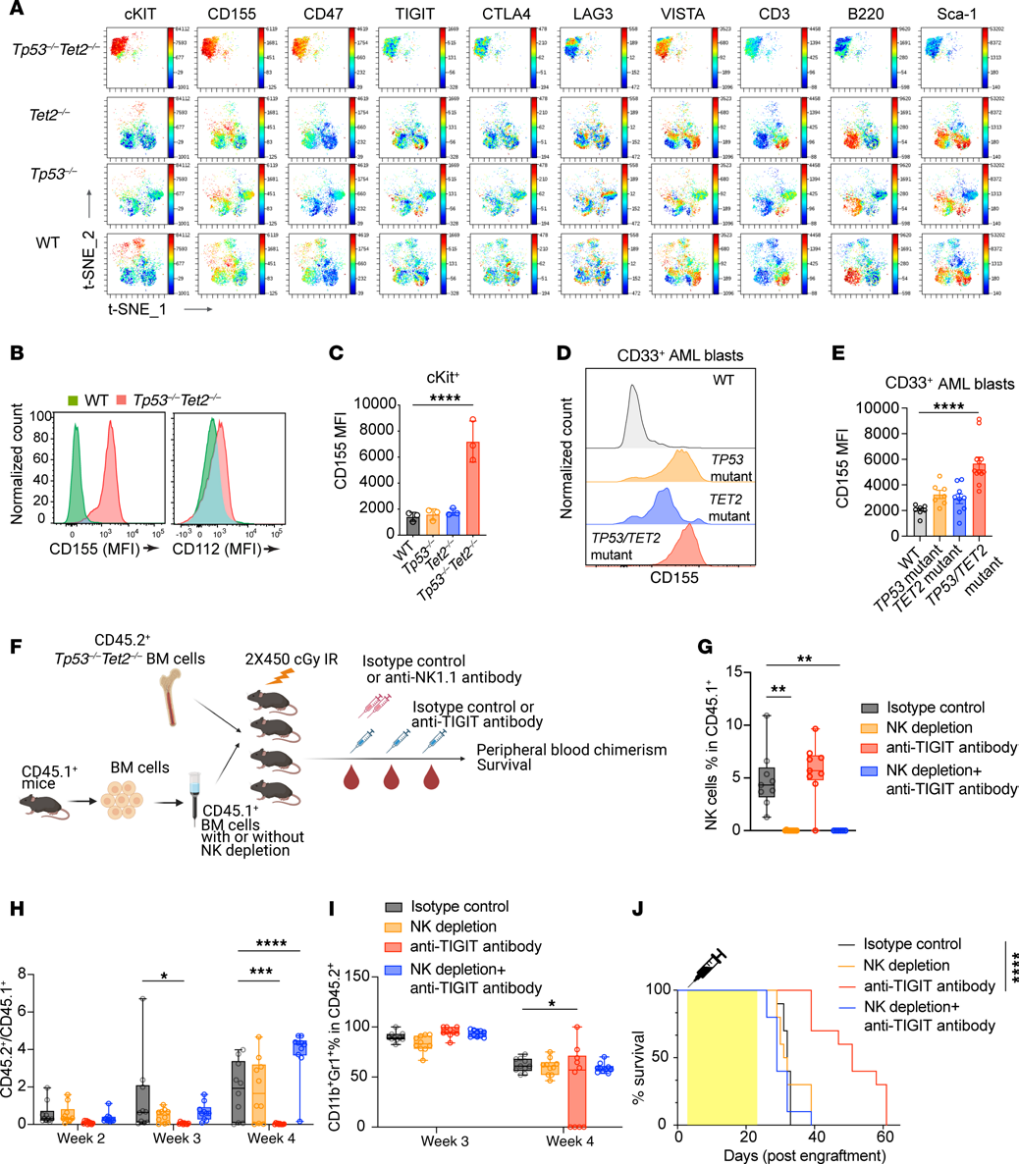
Immunosuppressive microenvironment of *TP53*/*TET2* double-mutant AML that is partially alleviated by TIGIT inhibition. (**A**) Representative t-distributed stochastic neighbor embedding (t-SNE) plots of multicolor flow cytometric analysis of selected immune checkpoint molecule levels on murine splenocytes from mice with the indicated genotypes. (**B**) Histograms of CD155 and CD112 expression by flow cytometry on LK cells of the bone marrow of *Tp53^–/–^Tet2^–/–^* or *Cre*^+^ control mice (WT). (**C**) Quantification of CD155 on murine cKIT^+^ progenitors of different genotypes by flow cytometry. Values (mean fluorescence intensity [MFI]) are shown as mean ± SEM; *n* = 3 mice/group; *P* values are shown (ANOVA with Dunnett’s test). ****P < 0.0001. (**D**) Representative flow plots and (**E**) quantification of CD155 expression on CD45^dim^SSC^lo^CD33^+^ blasts from patients with AML harboring different mutations by spectral flow. Values (mean fluorescence intensity MFI) are shown as mean ± SEM; *n* = 7–12 patients/group; *P* values are shown (ANOVA with Dunnett’s test). ****P < 0.0001. (**F**) Schematic of experiment to analyze the effect of NK depletion on anti-TIGIT antibody efficacy. (**G**) Box-and-whisker plots of percentage of NKp46^+^NK1.1^+^ cells in peripheral blood of mice at 2 weeks posttreatment initiation. **P < 0.01. (**H**) CD45.2^+^ (mutant) to CD45.1^+^ (WT) cell ratios and (**I**) CD11b^+^Gr1^+^ percentage in CD45.2^+^ cells in the peripheral blood of recipient mice. For box-and-whisker plots, boxes represent median, first, and third quartiles, with whiskers extending to 1.5× interquartile range. *P* values are shown (ANOVA with Dunnett’s test). *P < 0.05; **P < 0.01. ***P < 0.001; ****P < 0.0001. (**J**) Kaplan-Meier curves of lethally irradiated PepBoyJ CD45.1^+^ mice receiving whole or NK-depleted CD45.1^+^ supporting bone marrow cells and 1 × 10^6^ bone marrow cells from *Tp53^–/–^Tet2^–/–^* mice followed by IgG2a isotype control or anti-TIGIT antibody treatment. Log-rank test was used for survival statistics. ****P < 0.0001. *Tp53^–/–^Tet2^–/–^*, *Vav*-*cre* *Tet2^fl/fl^ Tp53^fl/fl^*; *Tet2^–/–^*, *Vav*-*cre* *Tet2^fl/fl^*; *Tp53^–/–^*, *Vav*-*cre* *Tp53^fl/fl^*; WT, *Vav-cre*.

To further evaluate the immune cell composition of *Vav*-*cre Tet2^fl/fl^* *Tp53^fl/fl^* mice, T, B, and NK cell identities were further classified using *ScType* (27). Cells consistent with naive B cells, immature B cells, and naive CD4^+^ T cells were present in AML (Supplemental Figure 9, A and B). In contrast to WT mice, effector CD8^+^ T, NK, and CD8^+^ NKT cells were depleted in AML mice (Supplemental Figure 9, C–E). Among T cell populations, a subset of *Cd3e^+^Tigit^+^Pd1^+^Lag3^+^* T cells, indicative of exhaustion, was identified in samples from *Vav*-*cre Tet2^fl/fl^* *Tp53^fl/fl^* AML mice (Supplemental Figure 9, F and G, and Supplemental Figure 10, A and B). These cells exhibited upregulation of the transcription factor, *Tox*, further supporting their exhausted phenotype in AML mice with *Tp53* and *Tet2* double-KO (Supplemental Figure 10, A and B) (28–30). Consistent with mouse scRNA-Seq, patient CITE-Seq analysis revealed that CD8^+^ T cells in *TP53* and *TET2* comutant patients exhibited increased expression of *TOX*, *CD244*, *TIGIT*, *TIM3*, and *LAG3*, which are key features of T cell exhaustion (Supplemental Figure 10, C and D). Bulk RNA-Seq of LK cells revealed genes encoding immune checkpoint receptors *Cd47*, *CD112*, and *Cd155*, among the top differentially expressed genes in *Vav*-*cre Tet2^fl/fl^* *Tp53^fl/fl^* AML. Flow cytometry confirmed that Cd155 and Cd47 were strongly expressed in cKIT^+^ leukemia progenitors from *Vav*-*cre Tet2^fl/fl^* *Tp53^fl/fl^* AML mice compared with cells from *Vav*-*cre Tet2^fl/fl^, Vav*-*cre Tp53^fl/fl^*, or WT mice (Figure 9, A–C, and Supplemental Figure 11A).

We next tested the relevance of upregulation of key immune checkpoint receptors identified above to human disease. We performed spectral flow cytometry on side scatter^lo^CD45^dim^CD33^+^ cells from patients with AML with *TP53/TET2* comutations (*n* = 12), *TET2* single mutations (*n* = 9), *TP53* single mutations (*n* = 7), or WT *TP53/TET2* (*n* = 7), separately. CD155 and CD47 were highly expressed on CD33^+^ AML cells (Figure 9, D and E, and Supplemental Figure 11, B–D). Malignant cells from patients with AML with combined *TET2* and *TP53* mutations had profoundly elevated CD155 expression compared with other groups (Figure 9, D and E; Supplemental Figure 11, B–D; and Supplemental Table 2). CD155 is a receptor for TIGIT on NK or NKT cells and a marker of CD8^+^ T cell dysfunction. CD112, also known as Nectin-2 or PVRL2, is an adhesion molecule of the Ig gene superfamily that plays a dual role in immune regulation. CD112 can costimulate T cell responses through CD226, while its interaction with inhibitory receptors like CD112R, TIGIT, and PVRIG suppresses T cell activation. The checkpoint receptor PVRIG competes with DNAM-1 for CD112 binding, and blocking PVRIG-CD112 interactions enhances T cell activation. In NK cells, CD112’s interaction with DNAM-1 promotes cytotoxicity, whereas its binding to TIGIT and PVRIG inhibits NK cell–mediated responses. In addition, TIGIT was upregulated in CD56^dim^CD16^+^ and CD56^hi^ NK cells in *TP53/TET2* comutant patient AML (Supplemental Figure 12, A–F).

We therefore hypothesized that elevated expression of CD112 and CD155 on AML blasts may contribute to NK or NKT immune evasion in the *TET2/TP53* double-mutant microenvironment. To evaluate the effect of the CD155-TIGIT interaction blockade on NK function, we cocultured murine NK cells with leukemic cells from *Vav*-*cre Tet2^fl/fl^* *Tp53^fl/fl^* mice in the presence or absence of anti-TIGIT antibody (Supplemental Figure 12G). Anti-TIGIT antibody augmented the ability of NK cells to kill *Tp53/Tet2* double-mutant AML. As a control, the isolated NK cells lysed prototypic tumor cell target Yac-1 cells at high efficiency (31). We next treated mice engrafted with *Vav*-*cre Tet2^fl/fl^* *Tp53^fl/fl^* AML cells with anti-TIGIT antibody or control. In this aggressive model, CD155 blockade significantly increased mouse survival and substantially reduced malignant cell burden in the animals receiving anti-TIGIT antibody (Supplemental Figure 12, H and I). Additionally, in anti-TIGIT antibody-treated spleens, there were more NK (CD3e^–^NK1.1^+^NKp46^+^) cells within the CD45.1^+^ tumor immune environment and concomitantly greater NK cell activation (as indicated by granzyme B expression) following anti-TIGIT treatment (Supplemental Figure 12, J and K).

We next determined the requirement of NK cells in response to anti-TIGIT antibody treatment (Figure 9F). Depletion of NK cells mitigated anti-TIGIT–mediated tumor suppression and attenuated anti-TIGIT–mediated inhibition of myeloid bias (Figure 9, G–I). In addition, anti-TIGIT–treated mice with NK depletion experienced anemia, thrombocytopenia, and leukocytosis comparable to isotype control-treated mice (Supplemental Figure 12, L and M). NK depletion completely abrogated the impact of anti-TIGIT antibody treatment on mouse survival (Figure 9J).

Interestingly, CD155 expression was also reduced on *Vav*-*cre Tet2^fl/fl^* *Tp53^fl/fl^* cells upon A20 depletion (Supplemental Figure 13, A and B). In parallel to the above, we engrafted CD45.2^+^ *Vav*-*cre Tet2^fl/fl^* *Tp53^fl/fl^* mouse AML cells, CRISPR edited with A20-targeting sgRNAs (sgA20#1 or sgA20#2) or nontargeting control sgRNAs, into lethally irradiated CD45.1^+^ recipient mice with supporting CD45.1^+^ bone marrow cells (Supplemental Figure 13C). Similarly to the in vitro results, A20 deletion reduced leukemia burden in vivo (Supplemental Figure 13D). Given that A20 deletion reduced CD155 expression, we tested the effect of anti-TIGIT antibody treatment in this model. A20 deletion resulted in even further reduction in *Vav*-*cre Tet2^fl/fl^* *Tp53^fl/fl^* leukemia burden and myeloid bias when coadministered with anti-TIGIT antibody (Supplemental Figure 13, D and E). Anti-TIGIT antibody treatment and A20 KO cooperatively extended the survival time of engrafted mice (Supplemental Figure 13F). These results reveal that AML-mediated NK dysfunction through CD155/TIGIT engagement contributes to AML driven by complete loss of *Tp53* and *Tet2*.

Altogether, these findings demonstrate that combined loss of *TP53* and *TET2* is associated with profound changes to the immune microenvironment, including increased expansion of monocytic MDSC-like cells, which suppress T cell activation in addition to driving T cell exhaustion and TIGIT-mediated evasion of NK cell–mediated killing.

## Discussion

Here, we show that *TP53* and *TET2* mutations cooperate to transform hematopoietic progenitors and play a role in development of AML. As such, these mice provide what we believe to be novel models of AML marked by clonal hematopoiesis and myeloid mutations. We found that hematopoietic progenitor cells in these models were marked by increased activation of innate immune signaling, and exposure of these mice to further environmental proinflammatory signals through TLR2 promoted noncanonical NF-κB pathway activation. These data support previous reports showing that aberrant TLR signaling in early hematopoietic progenitors is associated with a high risk of AML transformation (32) and that increased expression of TLR2 and signaling intermediates (like MyD88) is also seen in the bone marrow of patients with AML with no response to induction therapies compared with those that experience complete remission (32). This study utilized a mouse model with complete loss of *Tp53* and *Tet2* and therefore may not be representative of all forms of *TP53* mutations encountered in AML. As such it was important to evaluate many of the key findings from mice in patient samples with a diverse spectrum of *TP53* alterations seen in human AML.

The cooperative impact of *TP53* and *TET2* mutations in driving AML underscores the importance of understanding the temporal sequence of these mutations. Clonal hematopoiesis often arises from the acquisition of mutations in *TET2*, which may precede mutations in *TP53*. This sequence of events may create a proinflammatory environment that promotes the expansion of premalignant clones and transformation to AML. Conversely, *TP53* mutations occurring prior to *TET2* mutations could result in increased genomic instability and enhanced proliferation, with newly gained *TET2* mutations mediating a switch of lineage from lymphoid- to myeloid-biased leukemia. Future studies could leverage inducible mouse models to precisely control the timing and sequence of *Tp53* and *Tet2* mutations and provide further insight.

*TNFAIP3*, which encodes the A20 deubiquitinase that activates noncanonical NF-κB signaling, is frequently lost in lymphoma (33) where it promotes lymphomagenesis (33, 34). However, the role of A20 in AML has been less studied (35). A20 KO has been shown to decrease the competitive advantage of cKIT^+^ bone marrow cells in the TLR-TRAF6–overexpressing MDS mouse model (18). Consistent with these studies, we observed upregulation of A20 in response to TLR2 stimulation and a requirement for A20 in maintenance of *TP53/TET2* comutant AML. These data suggest that A20 activity may play roles in proinflammatory signaling and leukemic transformation in *TP53*/*TET2*-mutant AML in response to TLR2 signaling.

Beyond tumor-intrinsic effects, we found that *TP53* and *TET2* comutant AML in both patients and mice exists in an adaptive immune desert environment with reduced abundance of T, B, and NK cells. Moreover, T cells were exhausted in both mouse and human *TP53/TET2*-mutant AML, and there was clear accumulation of monocytic MDSCs that further suppressed T cell functions in *Vav*-*cre Tet2^fl/fl^* *Tp53^fl/fl^* mice. It will be interesting to explore the therapeutic potential of depleting monocytic MDSCs in the future, particularly in relation to their role in the maintenance of AML.

Upregulation of the immune checkpoints CD155 and CD47 was observed on cKit^+^ malignant cells from *Vav*-*cre Tet2^fl/fl^* *Tp53^fl/fl^* mice and in myeloid malignant cells from patients with *TP53/TET2* double-mutant AML. Moreover, A20 deletion downregulated the cell surface level of CD155. It is known that CD155 undergoes SUMOylation and inhibition of the SUMO pathway promotes CD155 translocation to the cell surface (36, 37). Therefore, it is possible that A20-mediated CD155 upregulation is dependent on A20-induced deubiquitination or indirect suppression of CD155 degradation. The relationship between A20 and CD155 warrants further investigation.

Upregulation of immune inhibitory checkpoints PD1, CTLA4, and CD47 has been consistently documented in myeloid neoplasms driving many prior clinical trials of antibodies aimed at blocking these proteins in patients with MDS and AML (38–42). Unfortunately, these studies have not yet yielded marked clinical benefits. Excitingly, here we propose a potential therapeutic strategy targeting TIGIT for the treatment of high-risk AML. CD155 interacts with TIGIT, an inhibitory receptor mainly expressed on NK, CD8^+^ T, and CD4^+^ T cells, thereby inhibiting the function of T and NK cells (43). Conversely, DNAM1 competes with TIGIT to engage with CD155 to activate NK cells. In support of this notion, CD155 has been reported as a negative prognostic marker for AML (44). We found that blocking the CD155-TIGIT interaction promoted NK cell–mediated killing of AML blasts and conferred survival benefit in AML-engrafted mouse models. We hope these findings will motivate future studies targeting the DNAM1 axis therapeutically in high-risk patients with AML who, based on our data, may likely benefit from TIGIT and CD155 blockade. Our study found overexpression of CD47 in double-KO mice and double-mutant patients, suggesting CD47’s role in immune evasion and leukemic cell survival in this genetic context. Despite CD47 being a promising therapeutic target, clinical trials for AML have shown mixed results. Magrolimab, a monoclonal antibody targeting CD47, demonstrated high response rates in phase Ib trials but was discontinued after the phase III ENHANCE-2 trial owing to lack of survival benefit and anemia-related toxicity (45–47). Other CD47-targeting agents like lemzoparlimab and evorpacept have been explored, with the former’s trials halted and the latter still in development (9, 48). Bispecific therapies like TG1801 showed initial promise but face limited development (48). These results raise questions about CD47 inhibition’s broader applicability in AML. However, it is possible that specific genetic subsets of *TP53*-mutant AML, such as those with concurrent *TET2* mutations, may still derive benefit from CD47-targeted therapies, particularly when combined with other immune-modulating strategies, such as TIGIT blockade. Future studies could explore the functional consequences of CD47 overexpression in this context and evaluate combinatorial approaches targeting multiple immune checkpoints.

## Methods

### Sex as a biological variable.

Both sexes were included in clinical sample analyses and animal studies to ensure comprehensive representation. The findings are anticipated to be applicable to both sexes.

### Patients.

Within the Alliance patient cohort of 1,603 adults and a University of Chicago cohort of 653 adults diagnosed with de novo AML, we identified 315 patients with *TP53* mutations, including 128 patients aged <60 years and 187 patients aged ≥60 years, who were treated on the Cancer and Leukemia Group B (CALGB)/Alliance frontline and University of Chicago treatment protocols between 2014 and 2021. Patients with acute promyelocytic leukemia were excluded. Patients were similarly treated with intensive cytarabine/daunorubicin-based induction chemotherapy and consolidation with high-dose chemotherapy or autologous HSC transplantation. Per study protocols, no patient received an allogeneic HSCT in first complete remission. All patients were enrolled on the CALGB 8461 (cytogenetic studies, clinical trial NCT00048958), CALGB 9665 (leukemia tissue bank, clinical trial NCT00899223), and CALGB 20202 (molecular studies) companion protocols.

### Mice.

Floxed homozygous *Tp53* (B6.129P2-Trp53tm1Brn/J, herein called *Tp53^fl/fl^*) mice were purchased from The Jackson Laboratory. *Vav-cre Tet2^fl/fl^* mice were donated by Ross Levine (Human Oncology and Pathogenesis Program, MSKCC, New York, New York, USA) (16). Peripheral blood was collected by retro-orbital bleeding in EDTA tube, and complete blood count analysis was performed using a Sysmex SN series analyzer (Sysmex Corporation) followed by differential counting. Mice were anesthetized with isoflurane and sacrificed by cervical dislocation upon meeting early removal criteria (e.g., weight loss, hind limb paralysis, lethargy, hunched posture, and difficulty breathing).

### Statistics.

Statistical analyses were performed using Prism 9.0 software. Mann-Whitney *U* test was performed between 2 nonparametric groups. For survival analysis, log-rank tests were used. Comparison of multiple groups was evaluated with 1-way ANOVA analysis followed by Dunnett’s test for multiple comparisons. *P* values of less than 0.05 were considered statistically significant.

### Study approval.

All procedures performed in the study followed the NIH *Guide for the Care and Use of Laboratory Animals* (National Academies Press, 2011) and were approved by the Committee on Ethics of Animal Experiments of The Ohio State University and Memorial Sloan Kettering Cancer Center. All patients provided study-specific written informed consent to participate in studies. Studies were performed in accordance with the Declaration of Helsinki and approved by institutional review boards of The Ohio State University, Memorial Sloan Kettering Cancer Center, and the University of Pennsylvania. All patient samples were collected in a pathologically annotated and deidentified fashion according to the guidelines of the ethics boards of each institute and center.

### Data availability.

Data are available in the Gene Expression Omnibus (GEO GSE217867, GSE285355, and GSE279386). Analysis code is publicly available at https://github.com/blaserlab/lapalombella_pu Values for all data points in graphs are reported in the Supporting Data Values file. Additional methods are provided in the Supplemental Methods.

## Author contributions

PZ, OAW, BWB, and RL conceptualized the study. PZ, MJC, SDB, OAW, BWB, and RL designed the study methodology. PZ, ECW, SJS, MG, CS, SAS, MC, XP, EDE, TL, BKH, WKC, Y Youssef, BC, AP, AML, CRC, NF, AC, JLL, SD, TK, ASM, DC, HP, KEW, JS, AL, TD, GL, CM, CJW, JSB, AFD, LA, RAB, Y. Yang, NRG, MJC, SDB, BWB, OAW, and RL performed experiments and analyzed data. PZ, AFD, OAW, BWB, and RL provided resources. PZ, OAW, BWB, and RL curated data. PZ, OAW, BWB, and RL wrote the original draft. PZ, OAW, BWB, and RL reviewed and edited the manuscript. PZ, OAW, BWB, and RL acquired funding. OAW, BWB, and RL supervised the study.

## Supplementary Material

Supplemental data

Unedited blot and gel images

Supplemental tables 1-7

Supporting data values

## Version 1 03/20/2025

In-Press Preview

## Version 2 05/15/2025

Electronic publication
