# Supplementary material for: TP53 mutations and TET2 deficiency cooperate to drive leukemogenesis and establish an immunosuppressive environment
Source: J Clin Invest. 2025 Mar 20;135(10):e184021. doi: 10.1172/JCI184021 (PMC12077897; doi:10.1172/JCI184021)
Supplement: Supplemental data [file jci-135-184021-s104.pdf]

SUPPLEMENTAL INFORMATION

Supplementary Materials and Methods

Mice

Mice were housed and maintained in a pathogen-free facility. Up to 5 mice were housed together in each cage and kept in a 12-hour light/dark cycle with the access to autoclaved food and water.

Floxed homozygous TP53 (B6.129P2-*Trp53*^{tm1Brn}/J, hereafter called *Tp53*$^{fl/fl}$) founder mice were purchased from the Jackson Laboratory (Bar Harbor, ME, USA). *Tet2*$^{fl/fl}$ *Vav-cre* mice were kindly donated by Dr. Ross Levine (1). Three weeks after birth, animals were weaned, and tails were harvested for genotyping. Peripheral blood was obtained from vein and collected in EDTA tube for monthly flow cytometry analysis and complete blood count (CBC) analysis using a Sysmex SN series analyzer (Sysmex Corporation) followed by differential counting. Mice were euthanized when they showed any signs of disease (e.g., weight loss, hind limb paralysis, lethargy, hunched posture and difficulty breathing). Mice were anesthetized with isoflurane and sacrificed by cervical dislocation. Blood and tissues were collected for being further processed for analysis. In selected experiments, single cell suspensions of blood, spleen and bone marrow were analyzed by flow cytometry for lineage markers as well as Sca-1 and cKIT upon live cell gating using BD Fortessa. The frequency of stem/progenitor cells were analyzed based on staining of markers (CD48, CD150, CD34 and CD16/32). CD45.1^{+} congenic C57BL/6J recipient mice were obtained from the Jackson Laboratory (stain # 002014). All mouse lines were maintained on a pure C57BL/6J genetic background (greater or equal to N10).

All procedures performed in the study followed the National Institute of Health Guide for the Care and Use of Laboratory Animals and were approved by the Committee on Ethics of Animal Experiments of Ohio State University and Memorial Sloan Kettering Cancer Center.

Cytogenetic and molecular genetic analyses of patient samples

Cytogenetic analyses of pretreatment bone marrow (BM) and/or blood samples subjected to short-term (24- or 48-h) unstimulated cultures were performed by CALGB/Alliance-approved institutional laboratories and the results confirmed by central karyotype review. The mutational status of 80 protein-coding genes was retrospectively determined centrally at the Clara D. Bloomfield Center for Leukemia Outcomes Research, The Ohio State University, by targeted amplicon sequencing using the MiSeq platform (Illumina, San Diego, CA), using DNA extracted from viably frozen cells collected via companion protocol CALGB 20202.

Immunohistochemistry (IHC) and histopathology

Full necropsy and gross examination of all tissues was performed. Tissues were fixed in 10% neutral buffered formalin, embedded in paraffin, sectioned at 5 mm onto glass slides and stained with hematoxylin and eosin by routine methodology. Bones were decalcified with 10% formic acid prior to paraffin embedding. Neoplasia in mice was assessed and classified according to the Bethesda Proposals For Classification of hematopoietic Neoplasia in Mice. For IHC staining, sections were hydrated and treated with citrate buffer pH 6.0 at 125°C for 30 sec. Rodent Block M (Biocare Medical, #RBM961) was applied, incubated for 20 min and rinsed. Staining was performed by sequential application and incubation with 1:25,000 anti-CD11b, 1:75 anti-Sca-1, 1:250 anti-CD45R, and anti-CD3 primary antibodies for 60 minutes at room temperature, rabbit on rodent HRP polymer (Biocare Medical, #RMR622) for 25 min at room temperature, DAB substrate (Biocare Medical, #BDB2004) for 5 min, and DAB Sparkle (Biocare Medical, #DS830). Hematoxylin (Biocare Medical, #CATHE) was used as a counter stain.

Flow cytometry

All flow data were acquired using BD Fortessa or Cytek Aurora. Results were analyzed with FlowJo software (Tree Star) or CytoBank (Beckman Coulter). t-distributed Stochastic Neighbor

Embedding (t-SNE) or uniform manifold approximation and projection (UMAP) were used for the visualization of spectral flow cytometry data.

Lineages of primary and engrafted cells were identified with markers: AF700-CD45.1 (A20), APC-Cy7-CD45.2 (104), PE-CD117 (2B8), FITC-CD11b (M1/70), APC-Gr-1 (R86-8C5), BUV395-B220 (RA3-6B2), PE-CF594-Sca-1(D7) and PerCPcy5.5-CD3 (17A2). Stem/progenitor cells were identified with markers: FITC-Lineage (a cocktail of CD3e, Ly6G/Ly6C, CD11b, CD45R/B220, TER119), PE-CD34 (RAM34), PE-CF594-Sca-1 (D7), PerCPcy5.5-CD48 (hm48-1), AF647-CD150 (Q38-480), BV650-CD117(2B8), PE-Cy7-CD16/32 (2.4G2) and APC-Cy7-CD45 (104). The immunophenotypic cell surface antigens are: LT-HSC (Lin^{-}cKIT^{+}Sca1^{+}CD48^{-} CD150^{+}); ST-HSC (Lin^{-} cKIT^{+} Sca1^{+} CD48^{-} CD150^{-}); multipotent progenitor (MPP; Lin^{-}cKIT^{+} Sca1^{+} CD48^{+} CD150^{-}); LSK (Lin^{-} cKIT^{+} Sca1^{+}); granulocyte macrophage progenitors (GMP; Lin^{-} cKIT^{+} Sca1^{-} CD16/32^{+} CD34^{+}); common myeloid progenitor (CMP; Lin^{-} cKIT^{+} Sca1^{-} CD16/32^{-} CD34^{+}); megakaryocyte-erythroid progenitors (MEP; Lin^{-} cKIT^{+} Sca1^{-} CD16/32^{-} CD34^{-}). For cell proliferative analysis, EdU(Life Technologies) was administrated as a single dose of 1 mg injected i.p. EdU incorporation was detected 16 hours later with Click-It EdU Alexa 488 Flow kit. Lin- cells were first enriched with lineage depletion kit. After Click-It reaction, cells were stained with antibody panels for GMPs and GMPs were sorted with BD Aria Fusion. Subsequently, the stained cells were analyzed by BD Fortessa.

Human immune checkpoint molecule expressions were identified with markers: FITC-VISTA (B7H5DS8), PE-CD34 (561), APC-Cy7-Lag3 (11C3C65), BV421-CD3 (UCHT1), APC-CTLA4 (BNI3), PE-Cy5.5-CD47 (CC2C6), PE-CY7-TIGIT (A15153G), BV605-CD155(SKII.4), BUV395-CD33 (WM53), BV786-CD45 (HI30), and BV711-PD1(EH12.2H7). Mouse immune checkpoint molecule expressions were identified with markers: FITC-CD11b(M1/70), PE-cKit(2B8), PE-CF594-Sca-1(D7), PerCP-Cy5.5-CD3e(145-2C11), PE-Cy7-NK1.1S17016D, APC-CTLA4(UC10-4B9), AF700-CD47 (miap301), BV421-CD134 (OX-86), BV510-

TIM3(B8.2C12), BV605-TIGIT (4D4mTIGIT), BV650-CD155 (TX56), BV711-LAG3 (C9B7W), BV786-VISTA (MIH63), BUV395-B220 (RA3-6B2), BUV737-PD1 (29F.1A12).

Human NK panel: V450-CD3 (UCHT1), PerCP-Cy5.5-CD16 (3G8), FITC-CD45 (HI30), APC-CD56 (QA17A16), and PE-TIGIT (Vstm3). Mouse NK activation panel: Pacific blue-CD3e (145-2C11), BV605-CD45.2 (104), APC-cy7-CD45.1 (A20), PerCP-Cy5.5-NK1.1 (S17016D), PE-NKp46 (29A1.4) and APC-granzyme B (QA16A02). For human and mouse immunephenotype profiling, antibodies were purchased from Biolegend or BD. MDSC cFluor spectral flow panel was obtained from Cytek.

CFU

10K bone marrow cells were plated in duplicate in semisolid medium MethoCult M3234 (Stem Cell Technologies) according to the manufacturer's protocol. The colonies were counted in a double-blinded manner after 7 to 14 days. Then, colonies were collected to repeat the same procedure for serial replatings.

Murine transplantation

Whole bone marrow competitive transplant: 1×10^6 bone marrow cells from *CD45.2*$^+$ *Vav-cre Tet2*$^{fl/fl}$ *Tp53*$^{fl/fl}$ (*Tp53*$^{-/-}$*Tet2*$^{-/-}$), *Vav-cre Tet2*$^{fl/fl}$ (*Tet2*$^{-/-}$), *Vav-cre Tp53*$^{fl/fl}$ (*Tp53*$^{-/-}$) and *Vav-cre (WT)* mice were injected into lethally irradiated CD45.1$^+$ recipient mice (2 X 450 cGy) with 1×10^6 CD45.1$^+$ supporting bone marrow cells. Chimerism and immune cell lineages were followed via FACS in the peripheral blood at indicated time points.

GMP transplant: Vav-cre Tet2$^{fl/fl}$ *Tp53*$^{fl/fl}$ (*Tp53*$^{-/-}$*Tet2*$^{-/-}$) mice at AML stage and age-matched *Vav-cre Tet2*$^{fl/fl}$ (*Tet2*$^{-/-}$), *Vav-cre Tp53*$^{fl/fl}$ (*Tp53*$^{-/-}$) and *Vav-cre (WT)* mice were used for this transplant. Bone marrow single cells suspension was prepared and GMPs (Lin$^-$cKit$^+$Sca-1$^-$CD16/32$^+$CD34$^+$) were enriched by FACS. 0.3×10^6 GMPs with 1×10^6 supporting CD45.1$^+$ bone

marrow cells were transplanted via tail-vein injection into lethally irradiated CD45.1$^+$ recipient mice.

NK depletion: Bone marrow cells from CD45.2$^+$ *Vav-cre Tet2$^{fl/fl}$ Tp53$^{fl/fl}$* mice were transplanted into lethally irradiated CD45.1$^+$ congenic recipient mice along with NK-depleted (using biotin anti-NK1.1 antibody and streptavidin beads; Miltenyi Biotec) or whole CD45.1$^+$ supporting bone marrow. One day later, recipient animals were then randomized to treatment with saline or NK depletion antibody (anti-NK1.1, clone PK136, BE0036, BioxCell) at 0.5 mg per dose i.p. at days, +4 and +7, followed by 10 mg/kg IgG2a isotype control or anti-TIGIT antibody (10A7-derived monoclonal antibody against murine TIGIT, mtigit-mab10-10, InvivoGen) three times a week for three weeks, administered i.v. first dose and subsequently i.p..

sgA20 and anti-TIGIT antibody treatment: 1X10^6 *Tp53$^{-/-}$Tet2$^{-/-}$* c-Kit+ bone marrow cells electroporated with sgNeg or sgA20-Cas9 RNP complex were engrafted into lethally irradiated CD45.1$^+$ mice along with CD45.1$^+$ supporting bone marrow cells. Subsequently, mice were treated with 10 mg/kg IgG2a isotype control or anti-TIGIT antibody (10A7-derived monoclonal antibody against murine TIGIT, mtigit-mab10-10, InvivoGen) three times a week for three weeks, administered i.v. first dose and subsequently i.p.. Chimerism and immune cell lineage and mouse survival were monitored.

Functional characterization of MDSC-like population

To characterize MDSC-like cells, spleen cells were stained for CD11b$^+$Ly6C^{lo}Ly6G$^+$ granulocytic MDSCs or CD11b$^+$Ly6C^{hi}Ly6G$^-$ monocytic MDSCs and analyzed based on previously described protocol (2). T cells were isolated using the Pan T cell isolation Kit (Stem cell technologies) and stained with 1.5 µM CFSE (Thermo Fisher Scientific, Karlsruhe, Germany) for 4min. For MDSC isolation, the Myeloid-Derived Suppressor Cell Kit (Stem cell technologies) was employed. For the assessment of T cell suppressive function of MDSCs, isolated MDSCs were co-cultured with

T cells. To this end, CFSE-labeled T cells from leukemia-naïve C57BL/6 mice were seeded into 12-well plates and co-cultured with MDSCs in the presence of anti-CD3/anti-CD28 mAb-coated beads (gibco®, Thermo Fisher Scientific, Karlsruhe, Germany) in RPMI 1640 medium supplemented with 10% FBS and IL-2 (10 IU/mL; PeproTech).

In selected conditions, $CD4^+$ and $CD8^+$ T cells were isolated using the EasySep mouse $CD4^+$ T cell isolation Kit (Stem cell technologies) and EasySep mouse CD8+ T cell isolation kit (Stem cell technologies) and stained with 1.5 μM CFSE. $CD4^+$ or $CD8^+$ T cells were cocultured with sorted monocytic MDSCs at 1:8 ratio in the presence or absence of blocking antibody, 10 μg/ml anti-IL-10 (InVivoMAb anti-mouse IL-10; BE0049, BioxCell), 10 μg/ml anti-TGFβ (InVivoMAb anti-mouse TGFb; BE0057, BioxCell), 50 μg/ml anti-PDL1 (InVivoMAb anti-mouse PD-L1 (B7-H1), BE0101, BioxCell) or 300 μM Nor-NOHA (Selleckchem) for 72 hours. After 72 hours, cells were harvested and CFSE dilution of T cells was analyzed by flow cytometry. Harvested cells were also stained with CD3-APC, CD4-APC-Cy7, and CD8-AF700 followed by intracellular staining with IFNγ-BUV395 and TNFα-PE-Cy7(Biolegend).

Rare-cell analysis of GMP, LK, and LSK populations of murine bone marrow

Limiting cell–RNA sequencing (LC-RNA-seq) was developed at Ohio State University and performed as described previously (3). Briefly, fresh murine bone marrow cells with different genotypes were isolated and a total of 500 viable GMP (lin^- $cKit^+$ $Sca1^-$ $CD16/32^+$ $CD34^+$), LK (Lin^- $cKit^+$ $Sca1^-$) and LSK (lin^- $cKit^+$ $Sca1^+$) from treated patient samples were sorted in triplicates directly into single-cell lysis buffer as the manufacturer’s protocol (#4458235 Thermofisher Scientific).

Cell lysates were use for reverse transcription using SuperScript VILO cDNA Synthesis Kit (#11754050 Thermofisher Scientific). Libraries were prepared using Ion AmpliSeq Transcriptom Mouse Gene Expression Panel (#A36553). Samples were barcoded to run

multiple libraries per sequencing chip (#4471250). Amplification was performed on ProFlex PCR system from Applied Biosystems by Lifetechnologies (#4484073). PCR product was purified with Agencourt AMPure XP kit (A63881 Beckman Coulter, Indianapolis, Indiana). Libraries were quantified using Qubit DNA HS Assay Kit (Q32851) and if necessary equalized using additional amplification step. Using Ion Library TAQMAN Quantitation kit (#4468802), and Agilent 2100 Bioanalyzer instrument with the Agilent High Sensitivity DNA kit (#5067-4626), libraries were proportionally diluted for template preparation. Template preparation was performed on OneTouch OT2 instrument with Ion 540 Kit OT2 kit (A27753), then enrich and purify Ion One Touch2 ES. Purified ISPs were run on Ion GeneStudio S5 Prime System (A38195), using 540 Kit OT2 (A27753) and Ion 540 Chip Kit (A27766).

Data were analyzed using the Torrent Server with Torrent Suite 5.18.1 version. Raw data were additionally aligned to the mm10 reference genome with TopHat2. Data processing was performed according to our previous described workflow, which identifies reliably quantifiable transcripts in low-input RNA-seq for differentially expressed gene (DEG) analysis. Differentially expression analysis was conducted with R Studio with DESeq2.

Mouse scRNA-seq analysis

$1X10^6$ Bone marrow cells from *Vav-cre Tet2*$^{fl/fl}$*Tp53*$^{fl/fl}$ mice developing AML as well as from *Vav-cre* control (WT) and *Vav-cre Tp53*$^{fl/fl}$ mice were engrafted into lethally irradiated 6–8-month PepboyJ mice with $1X10^6$ supporting CD45.1$^+$ cells. Bone marrow cells were harvested from recipient mice 3 weeks post-engraftment and subjected to 10X scRNAseq analysis.

A 10X Genomics Chromium machine was used for 3000–5000 single-cell capture. Cells were washed and partitioned into nanoliter-scale Gel Bead-In-Emulsions (GEMs), where all generated cDNA share a common 10x Genomics Barcode but uses a pool of ~750,000 barcodes to separately index each cell’s transcriptome. The silane magnetic beads and Solid Phase Reversible Immobilization beads were used to clean up the GEM reaction mixture and

the barcoded cDNA was then amplified in a PCR step. Libraries were prepared using the Chromium Single Cell 3′ Reagent Kits (v3.1): Single Cell 3′ Library & Gel Bead Kit v3.1 (PN-1000128), Single Cell 3′ Chip G Kit v3.1 (PN-1000127) and Single Index kit T Set A (PN-1000213) (10x Genomics). Libraries were sequenced on an Illumina Nova Seq to achieve 75 bp reads. The P7 and R2 primers were added during the GEM incubation and the P5 and R1 during library construction via end repair, A-tailing, adapter ligation and PCR. The final libraries contain the P5 and P7 primers used in Illumina bridge amplification. Following the Single Cell 3′ Reagent Kits (v3.1) user guide (manual part no. CG000204 Rev D), data was processed by the Cell Ranger pipeline (v6.0.2; 10x Genomics) using the mouse genome (GRCm38.p5). Read counts were normalized to library size (4), scaled by 10,000, log-transformed, and filtered. Quality control removed low-quality cells with a high percentage of reads mapped to mitochondrial genes (mito counts >2 median absolute deviations above the median) and/or low numbers of genes detected (feature counts > 500) using functions from *blaseRtools* (https://github.com/blaserlab/blaseRtools). The *Doubletfinder* package (v2.0.3) was used to remove high-likelihood doublets. UMAP dimensionality reduction, clustering, and top marker analysis was achieved using functions from *Monocle3* (v1.2.9) (5, 6). Cell clustering was performed using partition and Leiden clustering methods (7, 8). UMAP plots were generated utilizing *blaseRtools (9, 10)*.

Cellular Indexing of Transcriptomes and Epitopes by sequencing (CITE-seq) and Library Construction

Patient samples were thawed in prewarmed RPMI with 10% FBS. Viable cells were washed twice with cold PBS, labeled with TotalSeq-C-compatible Hashtags (BioLegend), and incubated with human Fc receptor-blocking agent (BioLegend). DAPI was used for live cell sorting via an Aria cytometer (BD Biosciences). After sorting, equal numbers of cells were washed once with

cold PBS, labeled with the TotalSeq-C Universal Cocktail (BioLegend), washed three more times with cold PBS, and submitted to the MSKCC Integrative Genomics Core for sequencing. A 10X Genomics Chromium 5' V3 technology was used for single-cell capture (3000–5000 cells). Cells were partitioned into nanoliter-scale Gel Bead-In-Emulsions (GEMs), where barcoded cDNA and antibody-derived tags (ADTs) were generated. The GEM reaction mixture was cleaned using silane magnetic beads and Solid Phase Reversible Immobilization beads, and cDNA/ADT libraries were amplified via PCR. Libraries were prepared using Chromium Single Cell 5′ Reagent Kits and sequenced on an Illumina NovaSeq to achieve 75 bp reads. Primers (P7 and R2) were incorporated during GEM incubation, while P5 and R1 were added during library construction. Final libraries contained P5 and P7 primers for Illumina bridge amplification.

CITE-seq Analysis

Samples were multiplexed into pools based on *TET2/TP53* genotype. A total of 30 patient samples (12 *TET2/TP53* co-mutant, 6 *TET2* mutant, 6 *TP53* mutant, and 6 wild-type) were processed. Sequencing data were pre-processed using Cell Ranger v8.0.0. Between 39,821 and 57,578 cells were identified per pool, with an average sequencing depth of 24,244 to 52,025 reads per cell.

Cell-feature barcode matrices were demultiplexed and processed using custom scripts (https://github.com/blaserlab/lapalombella.pu.datapkg/tree/master/inst/data-raw/cds_main_human_scripts). Quality control filtering excluded cells with low feature counts or high mitochondrial read percentages (11). A total of 207,670 cells remained in the dataset (range: 2–14,194 cells per sample). Gene expression UMI counts were normalized via log transformation and size-factor adjustment (5). ADT counts were normalized using Seurat's centered log ratio method. UMAP embeddings were generated without batch correction, and clustering and marker identification were performed using monocle3 and blaseRtools (10).

Mouse scRNA-seq data were projected onto human CITE-seq data using 1:1 mouse:human gene orthologs identified from the Alliance of Genome Resources database (12). Mouse gene identifiers were mapped to human orthologs, and projection into the UMAP space was performed with monocle3 (5). Processed data are available for download as an R package at https://figshare.com/s/e920d20f48d746004ff6. Code used for figure generation is available at https://github.com/blaserlab/lapalombella_pu. Raw data are deposited in GEO under accession GSE217867.

A20 knockdown

sgA20#1 (AACCATGCACCGATACACGC; crRNA), sgA20#2 (TCAACTGGTGTCGTGAAGTC;crRNA), and sgNeg (Negative Control crRNA #1, CAT#1072544) were synthesized from IDT (Integrated DNA Technologies, Inc.). Whole bone marrow cells were positively selected for c-Kit (CD117) using antibodies conjugated to magnetic beads (Miltenyi 130-091-224) and separated using MACS columns and separator (Miltenyi). C-Kit+ bone marrow cells were electroporated with sgRNA-Cas9 RNP at a cell density of 1×10^6. Electroporation was performed using the P3 Primary Cell 4D-Nucleofector L Kit (Lonza) following the manufacturer's instructions. crRNA and tracrRNA were annealed at 1:1 ratio. For each transfection, 2μl of 61μM Cas9 (IDT) and 1.4μl of 100μM annealed crRNA:tracrRNA (IDT) were mixed in 16.6μl of PBS at room temperature for at least 15 minutes to form RNP. c-Kit+ bone marrow cells were washed once with PBS, and resuspended in 80μl of P3 buffer containing 1.2μl Alt-R® Cas9 Electroporation Enhancer (IDT). The RNP mixture was added to the cells, and cells were electroporated using Lonza 4D-nucleofector X Unit with the "DK-100" program selected. Then, cells were subsequently cultured in Stemspan medium with supplements (StemCell Technologies) for 36 hours. Cells were then used for colony formation assay or transplanted into mice.

Western blotting

Mouse and human cells were lysed with IP lysis buffer for whole cell lysate. Nuclear extract was isolated with NE-PER Nuclear and Cytoplasmic Extraction Reagent (Thermo Scientific). Nuclear extracts and whole cell lysates were fractionated by 4 to 12% NuPAGE Bis-Tris Midi Protein Gels (Thermo Scientific), and then transferred onto a nitrocellulose membrane and blotted with indicated primary antibodies. Primary antibodies used in the study were obtained from Cell Signaling Technologies (anti-TLR2 (2229), anti-NIK Antibody (4994), anti-A20 (5630), anti-p100/p52 (4882), anti-Phospho-NF-κB2 p100 (Ser866/870) (4810), anti-RelB (4922), Phospho-IκBα (Ser32) (14D4) (2859), anti-IκBα (L35A5) (C4814), anti-NF-κB p65 (D14E12) (8242), anti-NF-κB1 p105/p50 Antibody (3035), anti-Phospho-IKKα/β (Ser176/180) (16A6) (2697), and anti-IKKα (3G12) (11930)) and Abcam (anti-Lamin B1 (ab16048)). Anti-β-actin−peroxidase antibody was obtained from Sigma (A3854). Blots were developed using either SuperSignal West Pico PLUS chemiluminescent substrate or SuperSignal West Femto Maximum Sensitivity substrate (Thermo Scientific).

Clinical endpoints and statistical analysis

CR required an absolute neutrophil count ≥1.5 x 10^9/L, a platelet count >100 x 10^9/L, absence of leukemic blasts in the blood or BM, cellularity greater than 20% with maturation of all cell lines, no Auer rods, less than 5% BM blasts, and no evidence of extramedullary leukemia, all of which had persisted for at least one month. OS was measured from the date on study until the date of death, and patients alive at last follow-up were censored. Data quality was ensured by review of data by the Alliance Statistics and Data Management Center and by the chairpersons of included studies following policies of the Alliance. Clinical and biological characteristics were compared using the Fisher's exact and Wilcoxon rank-sum tests for categorical and continuous variables, respectively. In our outcome analyses, we used *p*-values adjusted to control for per family error rate (probability of a Type I error) for all variables

considered in univariable analyses. For time-to-event analyses, we calculated survival estimates using the Kaplan-Meier method and compared groups using the log-rank test. Data collection and statistical analyses were performed by the Alliance Statistics and Data Management Center using SAS 9.4 and TIBCO Spotfire S+ 8.2. The median follow-up for patients still alive was 10 years.

Data sharing statement

Patient data used in survival analyses were obtained from the Alliance Statistics and Data Management Center, and the University of Chicago. Individual participant data will not be shared.

Supplementary Figures

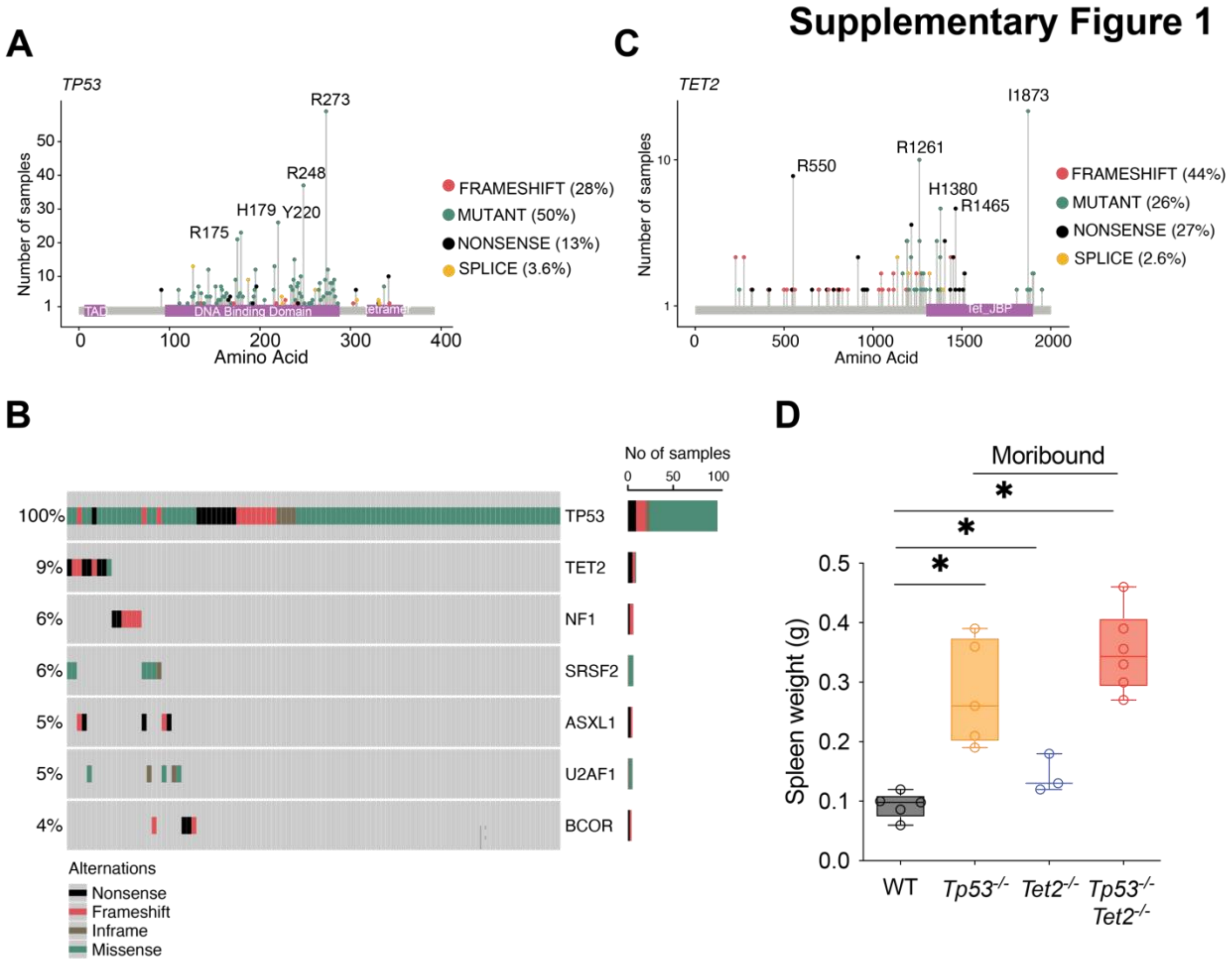

Supplementary Figure 1. Frequent co-occurrence of *TET2* and *TP53* mutations in AML and cooperativity of *Tet2* and *Tp53* deletion in mice. (A) Lollipop plot showing recurrent *TP53* mutations in leukemia samples from 3,723 patients (AACR GENIE). **(B)** Oncoprint of concurrent mutations in University of Chicago *TP53*-mutant patient cohort (99 patients). **(C)** Lollipop plot showing recurrent *TET2* mutations in leukemia samples from 668 *TP53*-mutant patients (AACR GENIE). **(D)** Box-and-whisker plots of spleen weights from 4-month-old mice with the indicated genotypes. Box and whisker plots: boxes represent median, first and third quartiles, with whiskers extending to 1.5× interquartile range. n=3-6 mice/group. $^*P<0.05$ (ANOVA with Dunnett's test). $Tp53^{-/-}Tet2^{-/-}$ = *Vav-cre* $Tet2^{fl/fl}$ $Tp53^{fl/fl}$; $Tet2^{-/-}$ = *Vav-cre* $Tet2^{fl/fl}$; $Tp53^{-/-}$ = *Vav-cre* $Tp53^{fl/fl}$; WT = *Vav-cre.*

Supplementary Figure 2

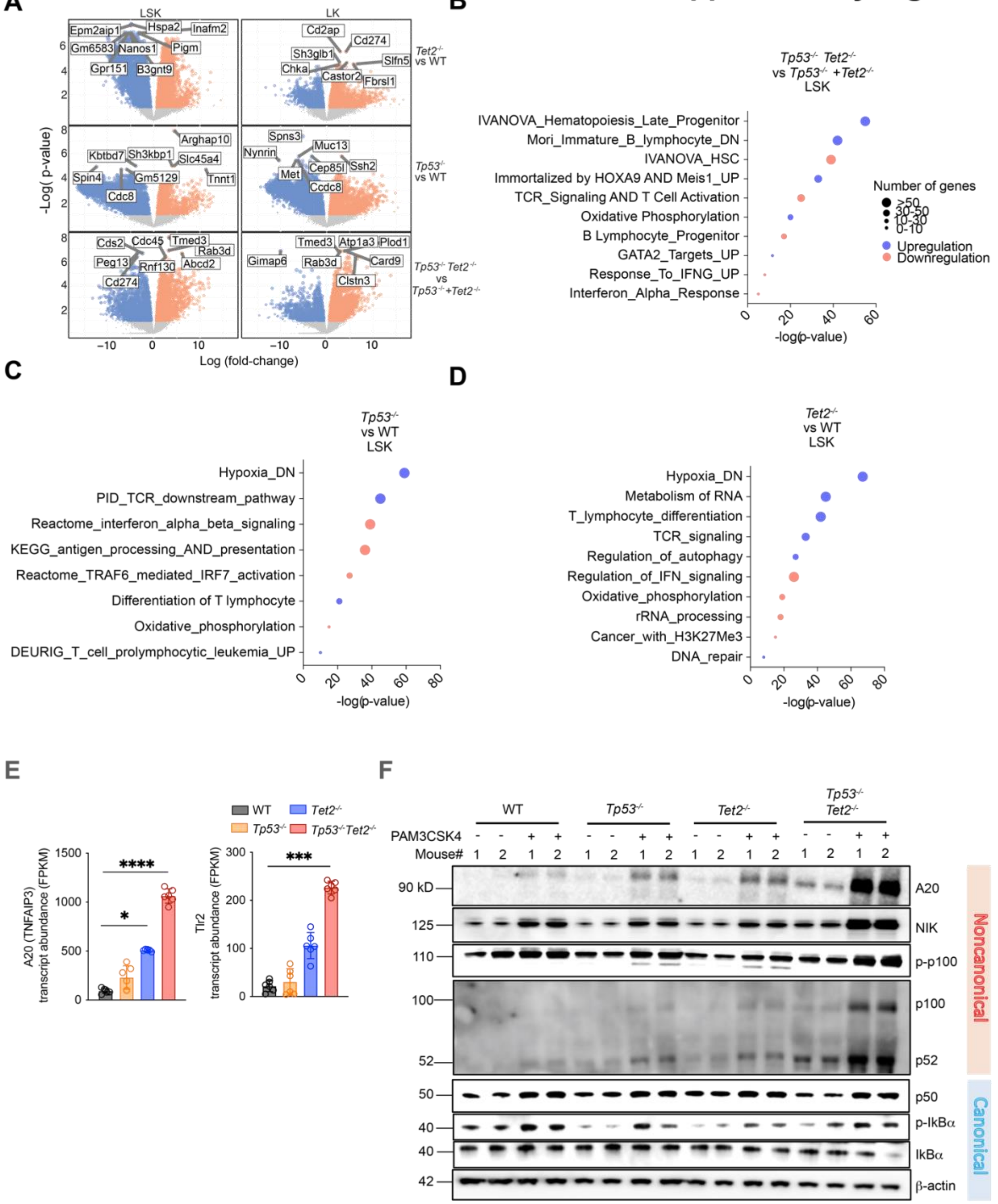

Supplementary Figure 2. Differentially expressed genes in mice with hematopoietic *Tet2* and/or *Tp53* deletion and upregulation of myeloid progenitor transformation pathways and innate immune signaling in *Tp53/Tet2* double knockout AML. (A) Volcano plots

depicting top differentially expressed genes in LSK and LK progenitor cells in different comparison groups. **(B)** GSEA pathway enrichment of top upregulated and downregulated pathways in *Vav-cre* $Tp53^{fl/fl}Tet2^{fl/fl}$ LSK cells. **(C)** GSEA pathway enrichment of top upregulated and downregulated pathways in *Vav-cre* $Tp53^{fl/fl}$ LSK cells. **(D)** GSEA pathway enrichment of top upregulated and downregulated pathways in *Vav-cre* $Tet2^{fl/fl}$ LSK cells. **(E)** Abundance of transcripts encoding *A20* (*Tnfaip3*) and *Tlr2* in LK cells from 4-month-old mice with the indicated genotypes. Mean$\pm$SD. n=5-6 mice/group. *P<0.05; *** P<0.001; **** P<0.0001. **(F)** Western blot of canonical and non-canonical NF-kB pathway components in $cKit^{+}$ bone marrow cells from age-matched $Tp53^{-/-}Tet2^{-/-}$, $Tet2^{-/-}$, $Tp53^{-/-}$ and WT mice in the presence or absence of 100 ng/ml PAM3CSK4 treatment for 12 hours. β-actin serves as housekeeping control. Results are representative of 2 independent experiments. $Tp53^{-/-}Tet2^{-/-}$ = *Vav-cre* $Tet2^{fl/fl}$ $Tp53^{fl/fl}$; $Tet2^{-/-}$ = *Vav-cre* $Tet2^{fl/fl}$; $Tp53^{-/-}$ = *Vav-cre* $Tp53^{fl/fl}$; WT = *Vav-cre*.

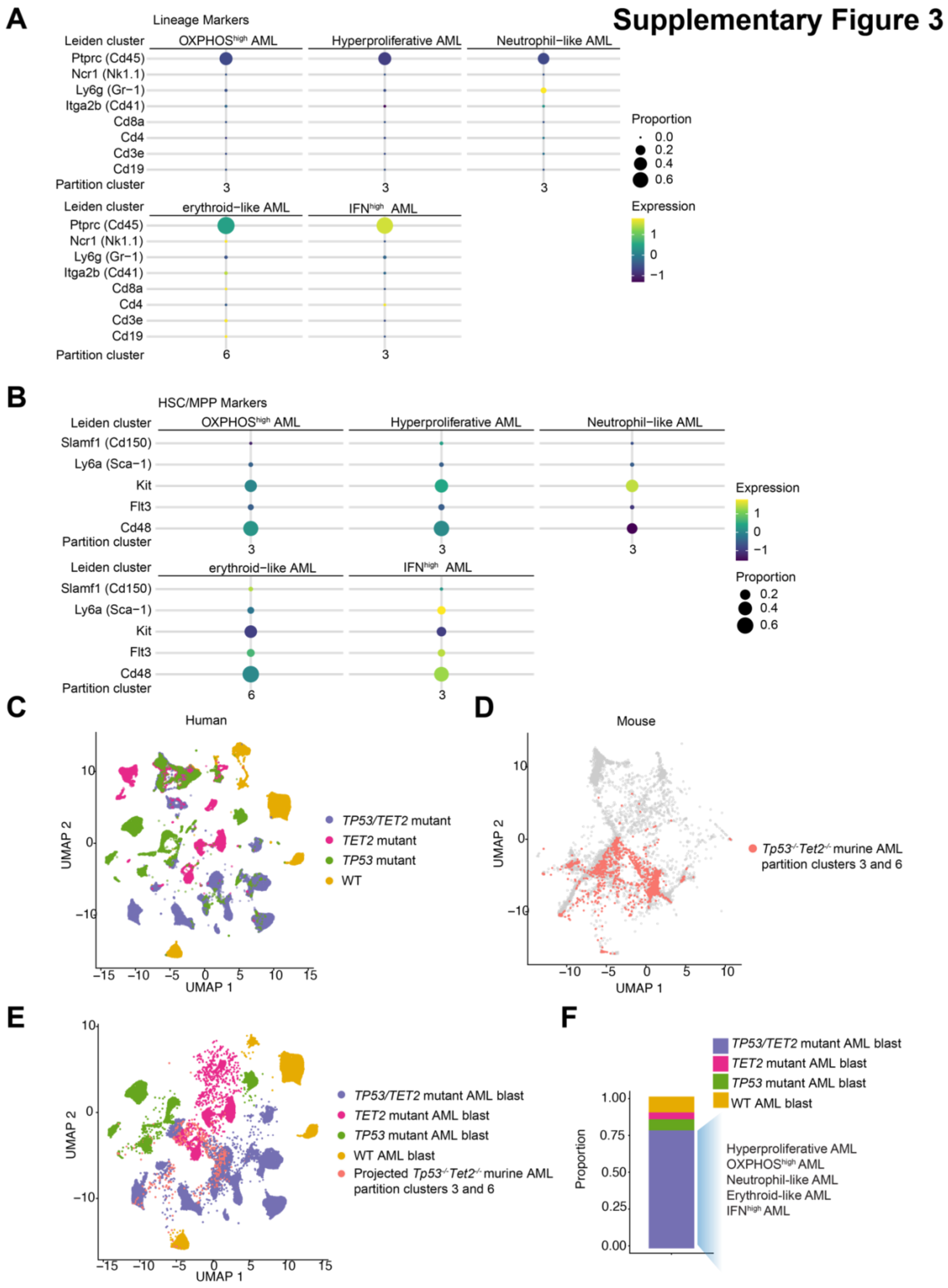

Supplementary Figure 3. Single cell transcriptomic signature of *Tp53/Tet2* double

knockout murine AML resembles human disease. (A) Bubble plots of lineage marker gene expression in clusters 3 and 6 and associated Leiden subclusters. **(B)** Bubble plots of HSC/MPP marker gene expression in clusters 3 and 6 and associated Leiden subclusters. The expression levels and proportions of selected markers are highlighted. UMAP representations of **(C)** human CITE-seq data visualizing human genotype clusters and **(D)** mouse scRNA-seq data highlighting murine clusters 3 and 6 (from Figure 6A) which are comprised solely of *Vav-cre Tet2*$^{fl/fl}$ *Tp53*$^{fl/fl}$ AML cells. CITE-seq data from 12 *TP53/TET2* mutant, 6 *TP53* mutant, 6 *TET2* mutant, and 6 WT AML patients (Table S2) and mouse scRNA-seq data are combined and undergo simultaneous dimension reduction analysis. **(E)** UMAP representations of combined human CITE-seq and mouse scRNA-seq data visualizing AML blast genotype-specific cluster regions. Human CD34^{+} AML blast clusters are shown after removal of normal human T-, B- and NK cells which none of mouse *Vav-cre Tet2*$^{fl/fl}$ *Tp53*$^{fl/fl}$ AML cells are mapped to. Projected *Vav-cre Tet2*$^{fl/fl}$ *Tp53*$^{fl/fl}$ AML cells (from Fig 6A: partition clusters 3 & 6) are highlighted. **(F)** Stacked bar chart illustrating the proportion of *Vav-cre Tet2*$^{fl/fl}$ *Tp53*$^{fl/fl}$ AML cells (from Figure 6A: partition clusters 3 & 6) that transcriptionally align with genotype-specific human AML.

Supplementary Figure 4

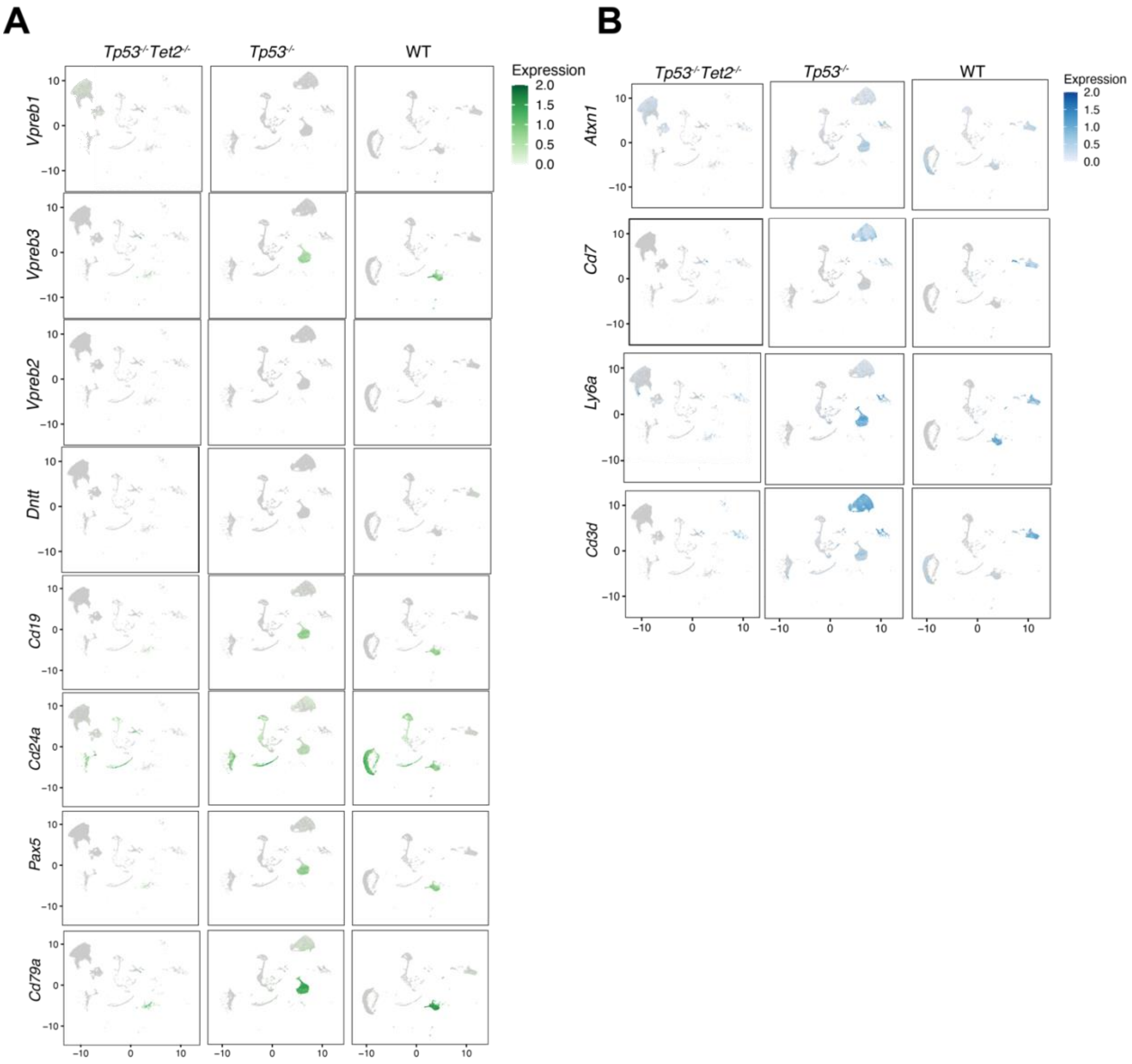

Supplementary Figure 4. **UMAP projection of single cells, colored by the expression of B and T cell markers. (A)** UMAP projection of single cells, colored by the expression of B- and pre-B cell markers. **(B)** UMAP projection of single cells, colored by the expression of T cell markers. *Tp53*$^{-/-}$*Tet2*$^{-/-}$ = *Vav-cre Tet2*$^{fl/fl}$ *Tp53*$^{fl/fl}$; *Tp53*$^{-/-}$= *Vav-cre Tp53*$^{fl/fl;}$ Wild type = *Vav-cre*.

Supplementary Figure 5

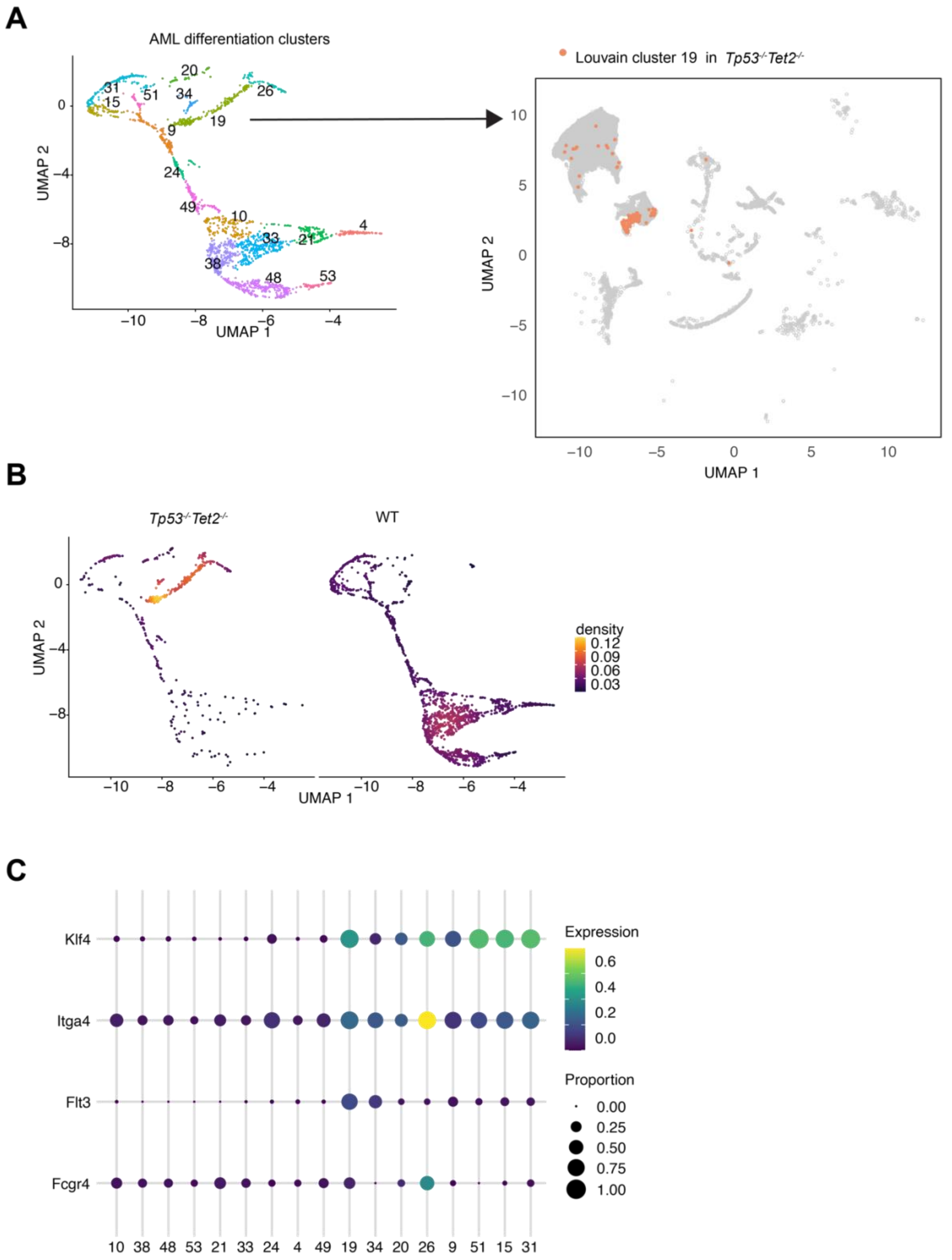

Supplementary Figure 5. Factors potentially regulating lineage commitment along

***TP53/TET2* co-mutant AML differentiation paths. (A)** Filtered Louvain clusters that are the potential location determining myeloid differentiation. Identification of cells found in Leiden cluster 19 in clusters 3 and 6 in Figure 6 in orange. **(B)** Cell density map showing cell populations are unique to *Vav-cre* $Tet2^{fl/fl}$ $Tp53^{fl/fl}$ AML phenotype. **(C)** Dot plots of top marker genes in Louvain clusters.

Supplementary Figure 6

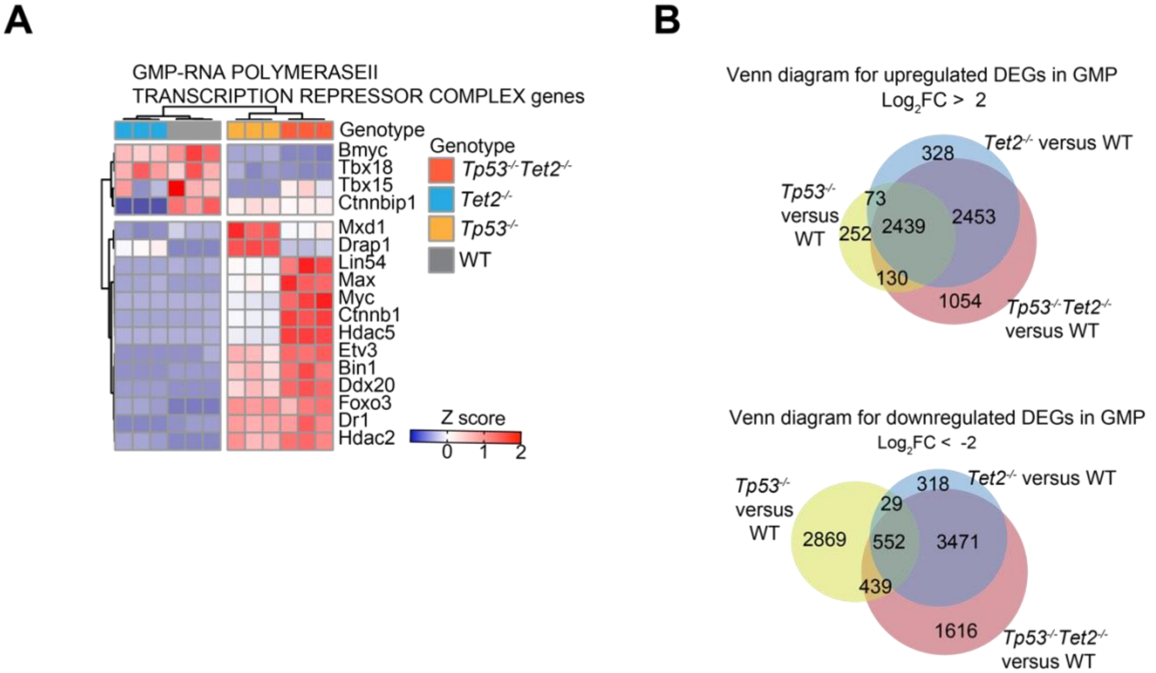

Supplementary Figure 6. Gene expression profile unique to *Tp53/Tet2* double knockout murine GMP. (A) Heatmap of top differentially expressed genes (adjusted *P-values*<0.05) within GOCC RNA POLYMERASEII TRANSCRIPTION REPRESSOR COMPLEX pathways of GMP cells from 4-month-old mice with different genotypes. Key genes upregulated in *Tp53*$^{-/-}$ *Tet2*$^{-/-}$ GMP cells are highlighted. **(B)** Venn diagram of shared top upregulated and downregulated differentially expressed genes (DEGs) in GMPs of double knockout and single knockout mice in relative to WT.

Supplementary Figure 7

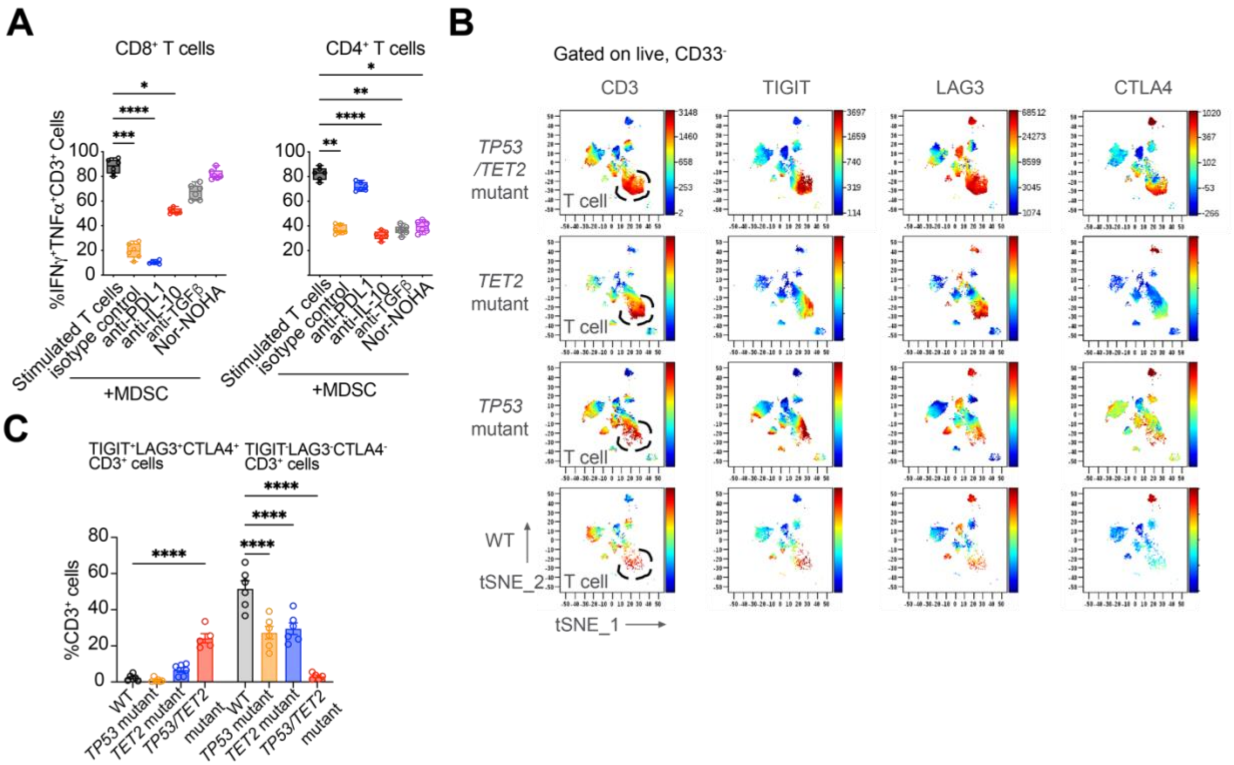

Supplementary Figure 7. Suppression of T cell activation by MDSCs from *Tp53/Tet2* double knockout murine and immune checkpoint expression on T cells in *TP53/TET2* co-mutant AML patients. (A) % activated T cells in T cell-MDSC coculture under different treatment conditions. Box and whisker plots: boxes represent median, first and third quartiles, with whiskers extending to 1.5X interquartile range. n=6 mice/group. *P-values* are shown (ANOVA with Dunnett's test). *$P<0.05$; **$P<0.01$; ***$P<0.001$; ****$P<0.0001$. **(B)** Representative UMAP plots of spectral flow cytometry analysis of the expression of selected immune checkpoint molecules on AML patient bone marrow samples gated on live $CD33^-$ cells. **(C)** Quantification of frequency of $TIGIT^+LAG3^+CTLA4^+$ T cells and $TIGIT^-LAG3^-CTLA4^-$ T cells among total $CD3^+$ T cells in patients with different mutations. Values are mean ± SEM; n=6 patients/group. *P*-values are shown (ANOVA with Dunnett's test). **** $P<0.0001$.

Supplementary Figure 8

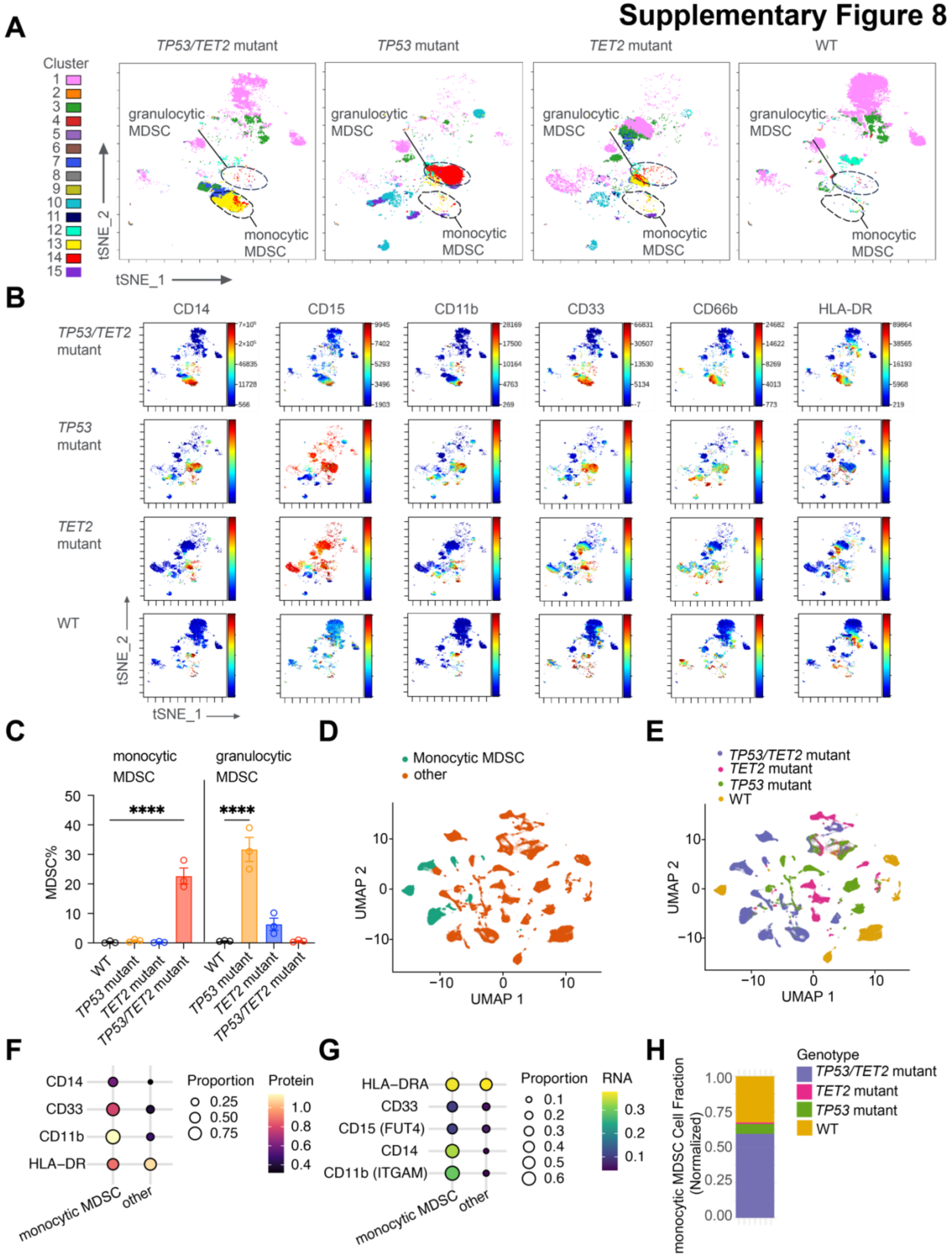

Supplementary Figure 8. The presence of monocytic MDSCs in *TP53* and *TET2* co-mutant

patients. (A) Spectral flow cytometric analysis of MDSCs in patients with *TP53* and *TET2* co-mutation, *TP53* mutation, *TET2* mutant or neither *TET2* or *TP53* mutations (WT). Leukemic blast, platelets, eosinophils or basophils are excluded from the analysis. Clusters 13 and 14 are monocytic MDSC ($CD33^+CD11b^+$ $HLA\text{-}DR^{low/-}$ $CD14^+CD15^-$ cells) and granulocytic MDSC ($CD66b^+CD33^{dim}CD11b^+$ $HLA\text{-}DR^-$ $CD14^-CD15^+$ subset), shown as circles, is highlighted in yellow and red. **(B)** Expression distribution of the indicated markers is shown for different patient genotypes. **(C)** Quantification of the percentage of monocytic and granulocytic MDSCs in non-$CD45^{dim}SSC^{low}$leukemic blast/platelet/eosinophil/basophil cells in in patients with different genotypes. Values are mean ± SEM; n=3 patients/group; *P*-values are shown (ANOVA with Dunnett's test). **** *P*<0.0001. UMAP representations of human AML CITE-seq data visualizing human cell type clusters **(D)** and patient genotype **(E)**. Monocytic MDSCs are identified as CD33 positive, CD11b positive, HLA-DR negative, CD14 positive and CD15 negative Leiden clusters. Other=cell types other than monocytic MDSC. CITE-seq data from 12 *TP53/TET2* mutant, 6 *TP53* mutant, 6 *TET2* mutant, and 6 WT AML patients (Table S2). **(F)** Bubble plots of MDSC marker protein expression. Antibody binding color scale is shown as the average Log_{10}-transformed protein binding (expression). Other=cell types other than monocytic MDSC. **(G)** Bubble plots of MDSC marker RNA expression. Expression color scale is shown as the average Log_{10}-transformed RNA level. Other=cell types other than monocytic MDSC. **(H)** Normalized monocytic MDSC cell fractions are stratified by AML patient genotype. Monocytic MDSC cluster fractions are normalized to account for variability in total cell counts across genotypes.

Supplementary Figure 9

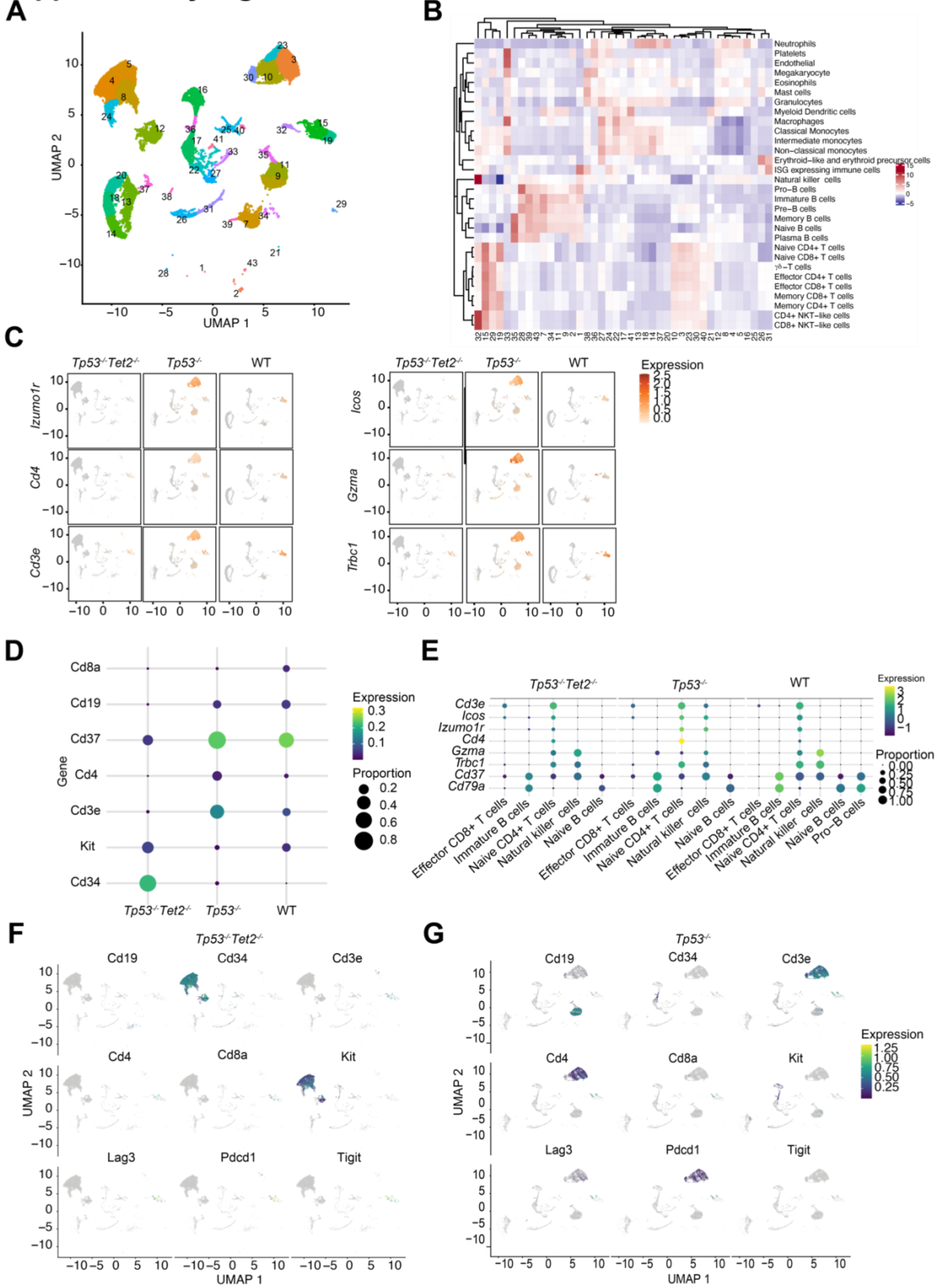

Supplementary Figure 9. Immune subtypes in *Vav-cre Tet2$^{fl/fl}$ Tp53$^{fl/fl}$* AML and *Vav*-cre

***Tp53*$^{fl/fl}$ mice. (A)** Immune cell populations in mouse bone marrow across different genotypes are annotated for Leiden clusters in UMAP space. The cell type scores were calculated for each Leiden cluster. The identities of the Leiden clusters were assigned by cell types with the highest calculated scores. **(B)** Heatmap of immune cell types in all annotated Leiden clusters. **(C)** UMAP of T cell gene expression across various genotypes **(D)** Dot plot of gene transcripts in the portion of cells and amounts in different genotypes. **(E)** Bubble plots of selected markers in different immune cell types across genotypes. The expressions of selected lineage marker and immune checkpoint genes of mouse **(F)** *Vav-cre Tet2*$^{fl/fl}$ *Tp53*$^{fl/fl}$AML and **(G)** *Vav-cre Tp53*$^{fl/fl}$ samples. *Tp53*$^{-/-}$*Tet2*$^{-/-}$ = *Vav-cre Tet2*$^{fl/fl}$ *Tp53*$^{fl/fl}$; *Tp53*$^{-/-}$ = *Vav-cre Tp53*$^{fl/fl;}$ WT = *Vav-cre*.

Supplementary Figure 10

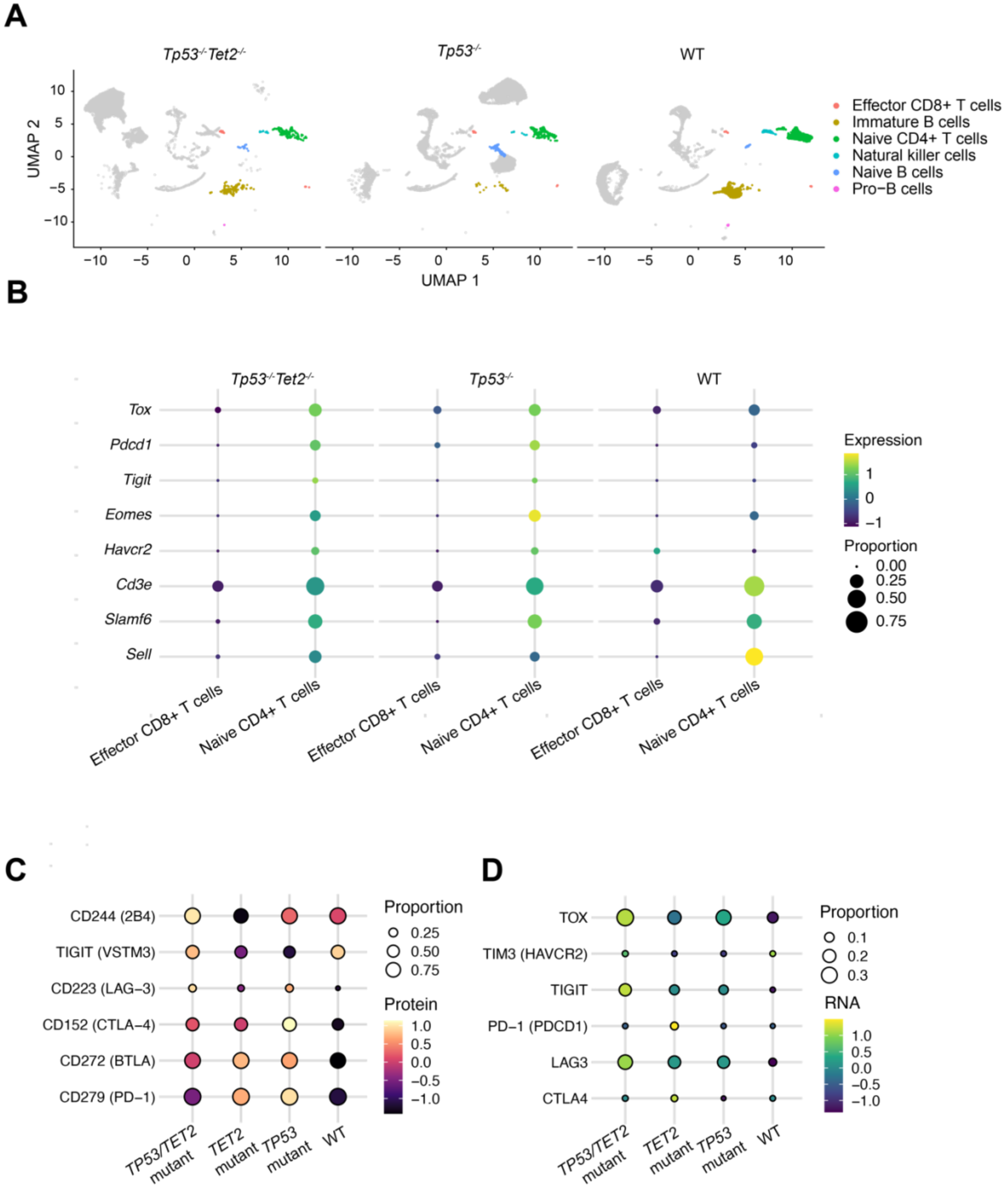

Supplementary Figure 10. Increased accumulation of exhausted T cells in murine and

human *TET2/TP53* double-mutant AML. (A) scRNA-seq analysis of subsets of T-, B-, and NK cells of murine bone marrow cells across different genotypes are annotated in Leiden clusters in UMAP space. The cell type scores were calculated for Leiden clusters. The identities of the Leiden clusters were assigned by cell types with the highest calculated scores. **(B)** T cells co-expressing *Cd3e, Tox, Eomes, Pdcd1, Tigit* and *Havcr2 (Tim3)* are present in *Vav-cre* $Tet2^{fl/fl}$ $Tp53^{fl/fl}$ AML tumor microenvironment. The expression levels and proportions of selected lineage markers and T cell exhaustion markers are highlighted in different Leiden clusters of T subsets of *Vav-cre* $Tet2^{fl/fl}$ $Tp53^{fl/fl}$ AML, *Vav-cre* $Tp53^{fl/fl}$ and WT bone marrow. $Tp53^{-/-}Tet2^{-/-}$ = *Vav-cre* $Tet2^{fl/fl}$ $Tp53^{fl/fl}$; $Tp53^{-/-}$ = *Vav-cre* $Tp53^{fl/fl}$; WT = *vav-cre.* **(C)** Bubble plots of T cell exhaustion marker protein expression on $CD8^{+}$ T cells stratified by patient genotype. Antibody binding color scale is shown as the average $\log_{10}$-transformed protein binding (expression). **(D)** Bubble plots of T cell exhaustion marker RNA expression on $CD8^{+}$ T cells stratified by patient genotype. Expression color scale is shown as the average Log_{10}-transformed RNA level. **(C)** and **(D)** are CITE-seq data from 12 *TP53/TET2* mutant, 6 *TP53* mutant, 6 *TET2* mutant, and 6 WT AML patients (Table S2).

Supplementary Figure 11

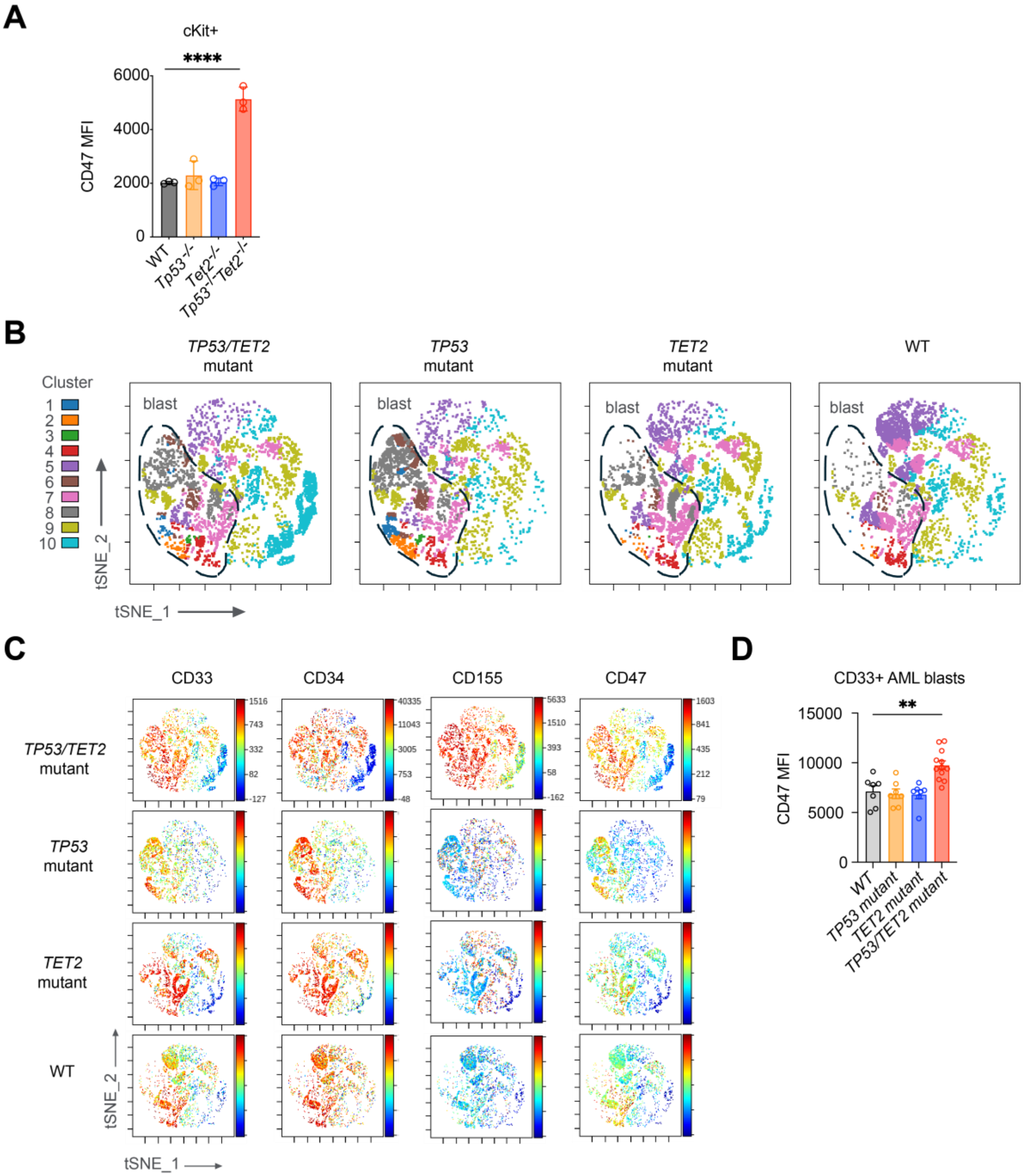

Supplementary Figure 11. Upregulation of immune checkpoint molecules on human *TET2/TP53* double-mutant AML cells and mouse *Vav-cre Tet2*$^{fl/fl}$ *Tp53*$^{fl/fl}$ AML cells. (A) CD47 on murine cKIT^{+} progenitors of different genotypes by flow cytometry. Values (mean fluorescence intensity MFI) are mean ±SEM; n=3 mice/group; *P*-values are shown (ANOVA with Dunnett's test). **** *P*<0.0001. **(B)** Spectral flow cytometric analysis of the expression of selected immune checkpoint molecules on AML patient bone marrow samples gated on CD45^{dim}SSC^{low}cells. Blast clusters are demarcated. **(C)** Expression distribution of the indicated

markers is shown for different patient genotypes. **(D)** Quantification of CD47 on $CD45^{dim}SSC^{low}CD33^{+}$ cells from AML patients harboring different mutations by spectral flow. Values (mean fluorescence intensity MFI) are mean ±SEM; n=7-12 patients/group; *P*-values are shown (ANOVA with Dunnett's test). ***P*<0.01.

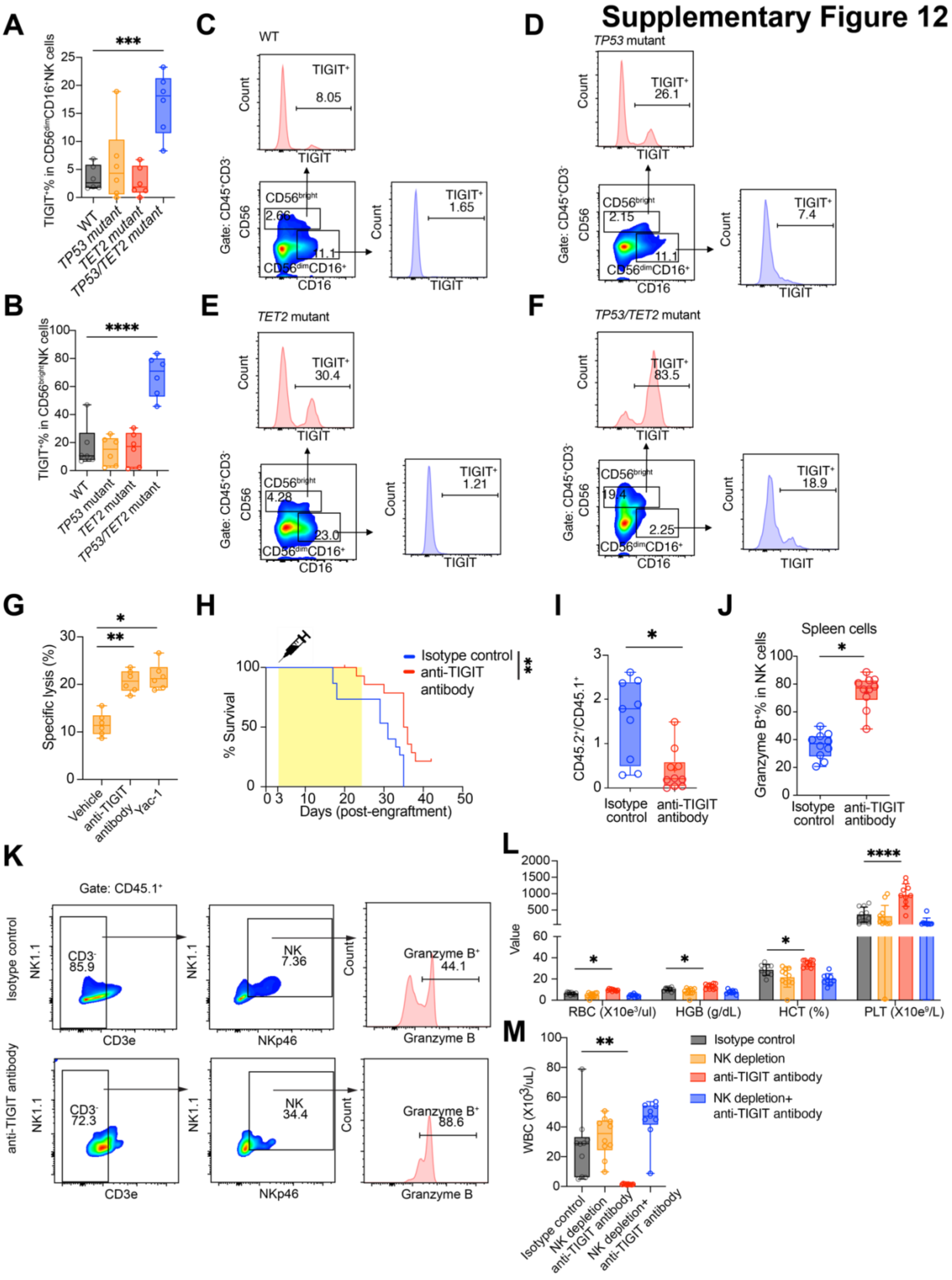

Supplementary Figure 12. Anti-TIGIT-mediated anti-tumor effect is dependent on NK

activation. Frequency of TIGIT$^+$ cells in **(A)** CD56^{dim}CD16$^+$ and **(B)** CD56^{high} NK subsets in AML patient samples (n=6). **(C-F)** Representative flow plots showing gating strategies in AML patient samples with the indicated genotypes. **(G)** Box-and-whisker plots of percentage of cell lysis from a 4-hour adenylate kinase release assay with NK cells isolated from spleens of wild-type mice cultured in IL-2 (90 ng/ml) and anti-TIGIT mAb (clone 10A7) or vehicle and exposed to *Tp53*$^{-/-}$ *Tet2*$^{-/-}$ cKIT$^+$ AML cells. Anti-mouse lymphocyte rabbit Ab (Accurate Chemical) coated cKIT$^+$ cells served as targets, with Yac-1 cytotoxicity as a positive control. **(H)** Kaplan-Meier curves of CD45.1$^+$ mice receiving 1X10^6 *Tp53*$^{-/-}$*Tet2*$^{-/-}$ bone marrow cells followed by isotype control or anti-TIGIT antibody. The treatment scheme is 10 mg/kg isotype control or anti-TIGIT mAb (clone 10A7) 3x/week for 3 weeks (i.v. first, then i.p.). A log-rank test was used for survival statistics. **(I)** Box-and-whisker plots of CD45.2$^+$ (mutant) to CD45.1$^+$ (WT) cell ratios in the spleens following 20 days of indicated treatment. **(J)** Box-and-whisker plots of granzyme B+ NK cells (percentage of CD45.1$^+$ NK cells) at endpoint splenocytes. Treated CD45.1$^+$ mice engrafted with CD45.2$^+$ *Vav-cre Tet2*$^{fl/fl}$ *Tp53*$^{fl/fl}$ AML were euthanized at day 20. Granzyme B$^+$ CD45.1$^+$ CD3e$^-$ NK1.1$^+$ NKp46$^+$ splenocytes were detected and quantified (n=10, *P<0.05 Mann-Whitney U test). **(K)** Representative flow plots of NK activation. **(L)** CBC parameters (mean ± SEM), and **(M)** total WBC count were quantified in peripheral blood of recipient mice at week 3. CD45.1$^+$ mice receiving whole or NK-depleted CD45.1$^+$ supporting bone marrow cells and 1X10^6 *Tp53*$^{-/-}$*Tet2*$^{-/-}$ bone marrow cells, followed by indicated treatment. Box and whisker plots show median, Q1-Q3, and whiskers at 1.5× IQR. *P*-value from ANOVA with Dunnett's test. *P<0.05; **P<0.01. ***P<0.001; **** P<0.0001. RBC=red blood cells; HGB=hemoglobin; HCT=hematocrit; PLT=platelets.

Supplementary Figure 13

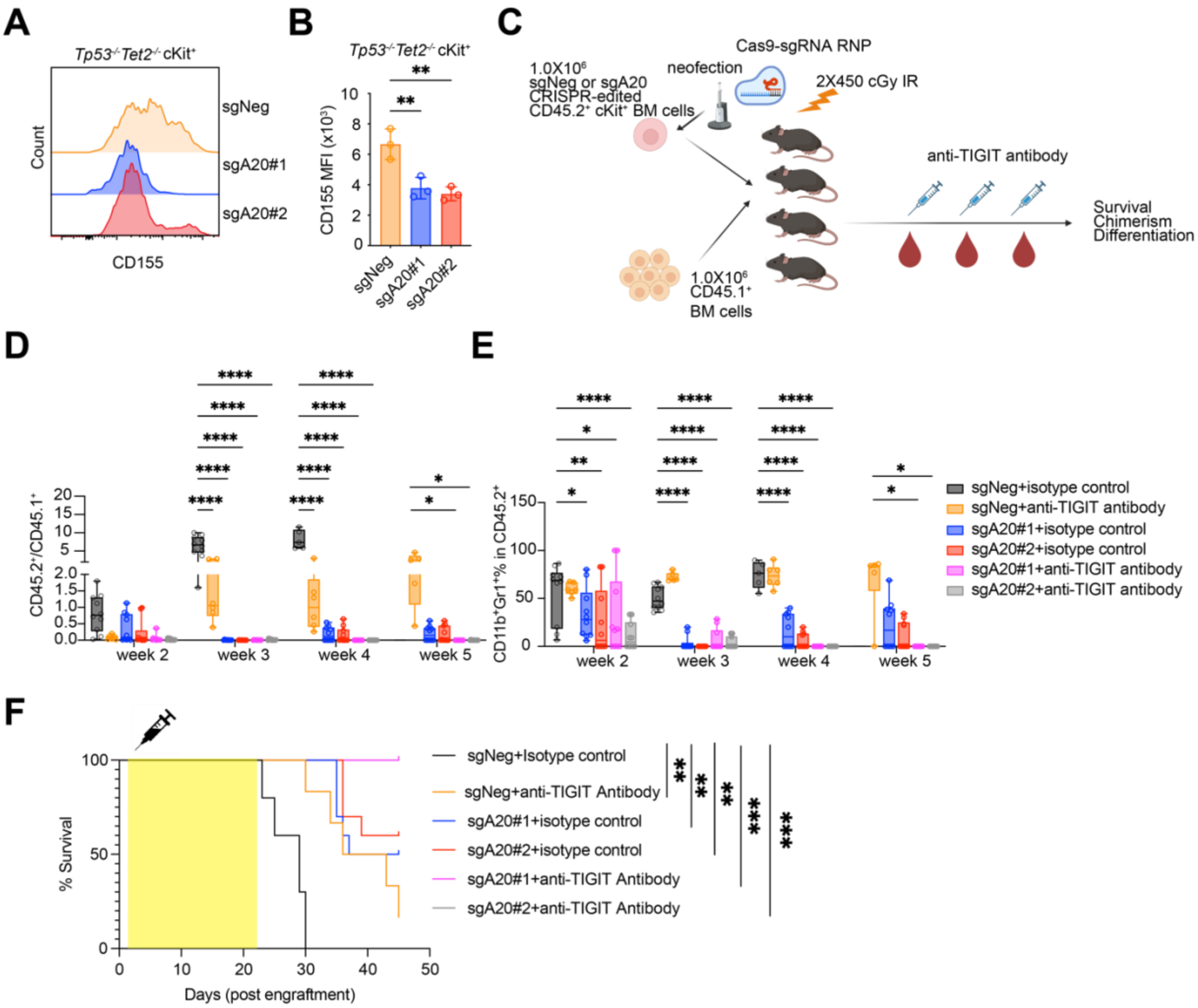

Supplementary Figure 13. A20 is required for maintenance of *Tp53/Tet2* double knockout AML and A20 depletion augments the anti-tumor efficacy of TIGIT blockade. (A) Representative flow plot and **(B)** quantification of CD155 on murine cKIT$^+$ progenitors by flow cytometry. Values (mean fluorescence intensity MFI) are mean±SEM; n=3 mice/group. *P-values* are shown (ANOVA with Dunnett's test). ***P*<0.01**. (C)** Schematic of in vivo study of anti-TIGIT antibody treatment and A20 depletion in recipient mice engrafted with *Tp53$^{-/-}$ Tet2$^{-/-}$* AML. 1X10^6 *Tp53$^{-/-}$Tet2$^{-/-}$* cKit$^+$ bone marrow cells with sgNeg or sgA20 were engrafted into CD45.1$^+$ mice along with CD45.1$^+$ supporting bone marrow cells. Mice were treated with 10 mg/kg IgG2a isotype control or anti-TIGIT mAb (clone 10A7) three times a week for three weeks, administered i.v. first dose and subsequently i.p.. Chimerism, lineage and mouse survival were monitored. **(D)** Box-and-whisker plots of CD45.2$^+$ (mutant) to CD45.1$^+$ (WT) cell ratios. **(E)** Percentage of CD11b$^+$Gr1$^+$ cells within CD45.2$^+$ cells in the peripheral blood of recipient mice. Box and whisker plots: boxes represent median, first and third quartiles, with whiskers extending to 1.5× interquartile range. *P-values* are shown (ANOVA with Dunnett's test). **P*<0.05; **** *P*<0.0001. **(F)** Kaplan-Meier curves of recipient mice. Log-rank test was used for survival statistics. ***P*<0.01; ****P*<0.001. *Tp53$^{-/-}$Tet2$^{-/-}$ = Vav-cre Tet2$^{fl/fl}$ Tp53$^{fl/fl}$.*

REFERENCES

1. Moran-Crusio K, Reavie L, Shih A, Abdel-Wahab O, Ndiaye-Lobry D, Lobry C, et al. Tet2 loss leads to increased hematopoietic stem cell self-renewal and myeloid transformation. *Cancer cell.* 2011;20(1):11-24.
2. Bronte V, Brandau S, Chen SH, Colombo MP, Frey AB, Greten TF, et al. Recommendations for myeloid-derived suppressor cell nomenclature and characterization standards. *Nat Commun.* 2016;7:12150.
3. Hu EY, Blachly JS, Saygin C, Ozer HG, Workman SE, Lozanski A, et al. LC-FACSeq is a method for detecting rare clones in leukemia. *JCI Insight.* 2020;5(12).
4. Hao Y, Hao S, Andersen-Nissen E, Mauck WM, Zheng S, Butler A, et al. Integrated analysis of multimodal single-cell data. *Cell.* 2021;184(13):3573-87.e29.
5. Trapnell C, Cacchiarelli D, Grimsby J, Pokharel P, Li S, Morse M, et al. The dynamics and regulators of cell fate decisions are revealed by pseudotemporal ordering of single cells. *Nat Biotechnol.* 2014;32(4):381-6.
6. Cao J, Spielmann M, Qiu X, Huang X, Ibrahim DM, Hill AJ, et al. The single-cell transcriptional landscape of mammalian organogenesis. *Nature.* 2019;566(7745):496-502.
7. Wolf FA, Hamey FK, Plass M, Solana J, Dahlin JS, Göttgens B, et al. PAGA: graph abstraction reconciles clustering with trajectory inference through a topology preserving map of single cells. *Genome Biol.* 2019;20(1):59.
8. Traag VA, Waltman L, and van Eck NJ. From Louvain to Leiden: guaranteeing well-connected communities. *Sci Rep.* 2019;9(1):5233.
9. Hing ZA, Walker JS, Whipp EC, Brinton L, Cannon M, Zhang P, et al. Dysregulation of PRMT5 in chronic lymphocytic leukemia promotes progression with high risk of Richter's transformation. *Nat Commun.* 2023;14(1):97.
10. Binder V, Li W, Faisal M, Oyman K, Calkins DL, Shaffer J, et al. Microenvironmental control of hematopoietic stem cell fate via CXCL8 and protein kinase C. *Cell Rep.* 2023;42(5):112528.
11. McCarthy DJ, Campbell KR, Lun AT, and Wills QF. Scater: pre-processing, quality control, normalization and visualization of single-cell RNA-seq data in R. *Bioinformatics.* 2017;33(8):1179-86.
12. Alliance of Genome Resources C. Updates to the Alliance of Genome Resources central infrastructure. *Genetics.* 2024;227(1).
