# Supplementary figures and images for: TP53 mutations and TET2 deficiency cooperate to drive leukemogenesis and establish an immunosuppressive environment
Source: J Clin Invest. 2025 Mar 20;135(10):e184021. doi: 10.1172/JCI184021 (PMC12077897; doi:10.1172/JCI184021)

WCL

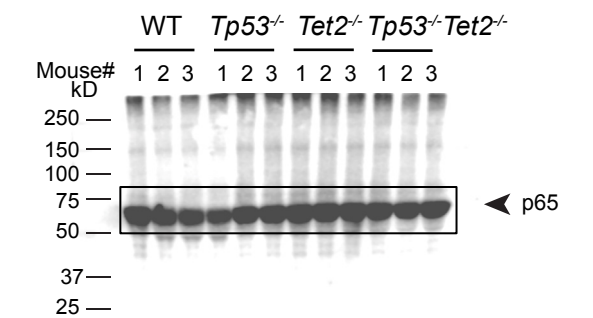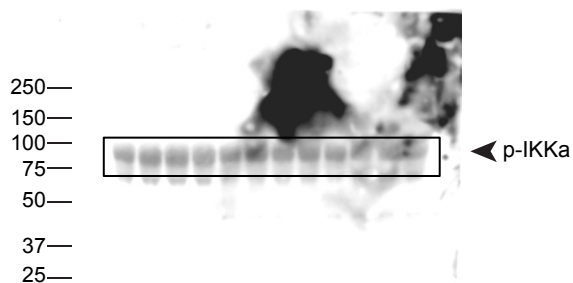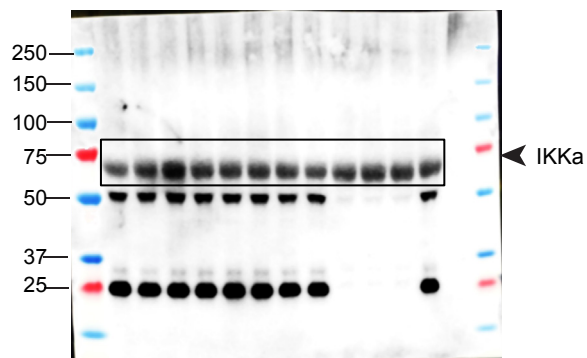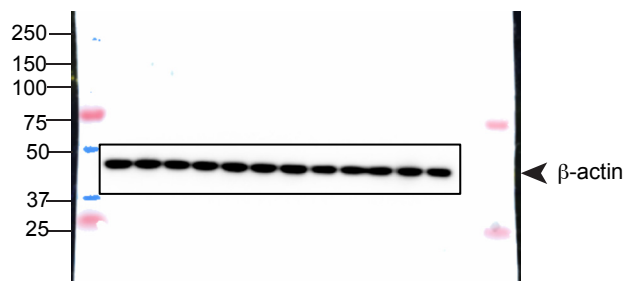

NE

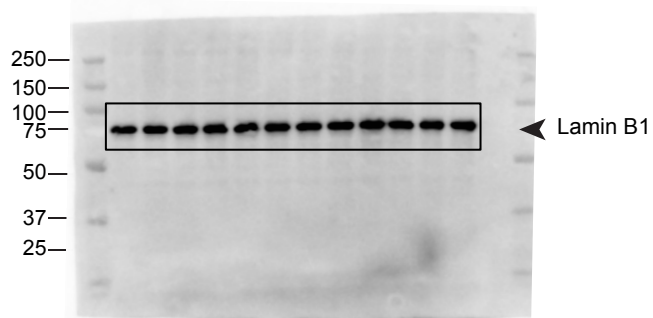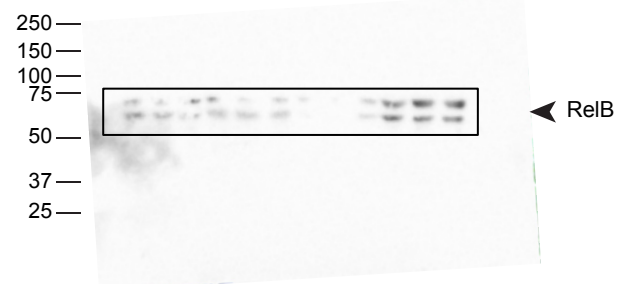

WCL

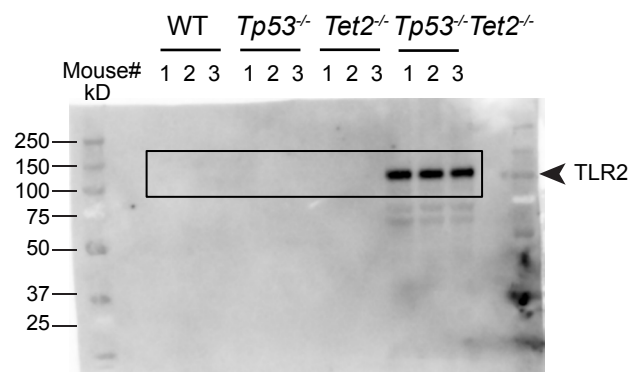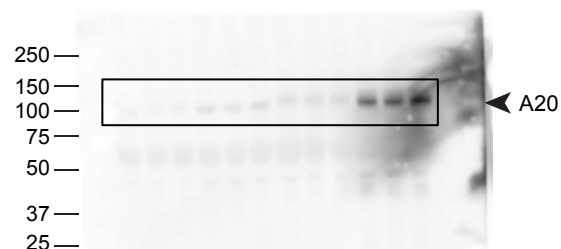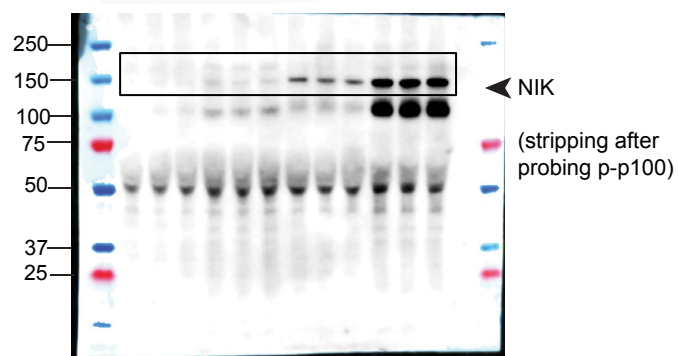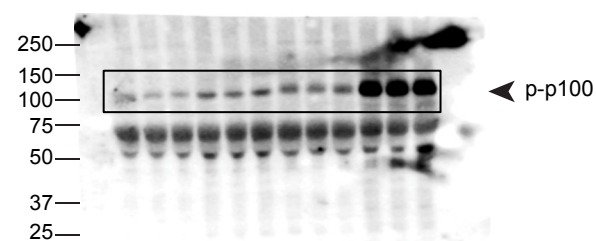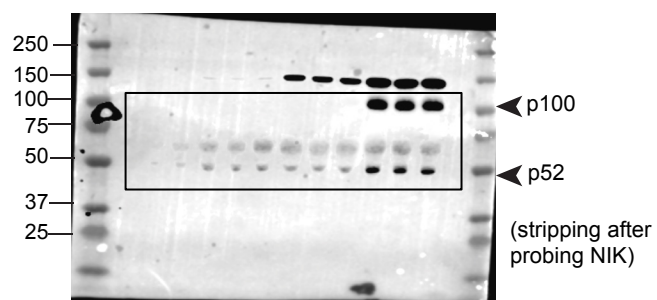

Full unedited blots for Figure 5C

NE

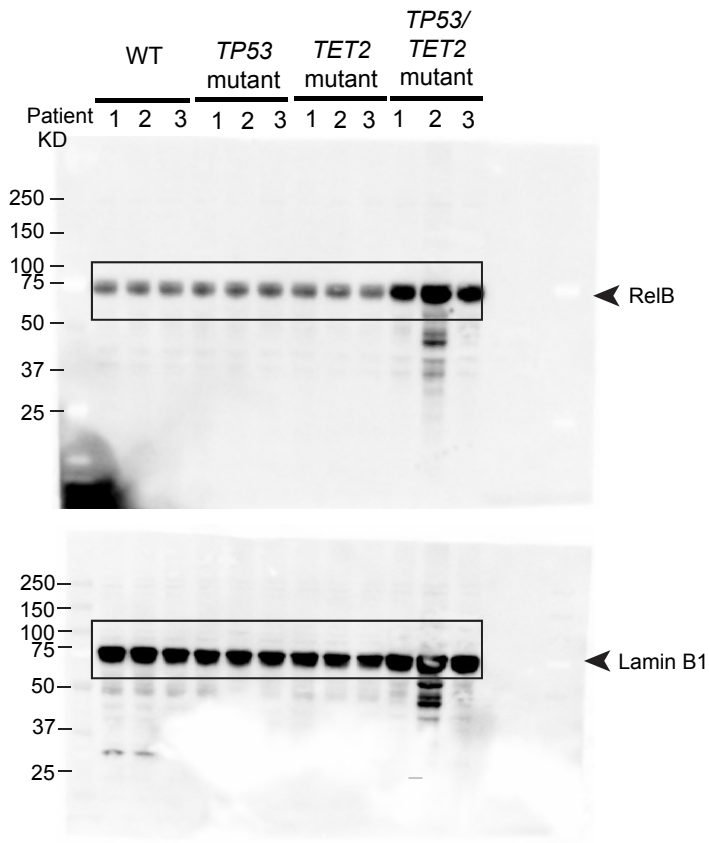

WCL

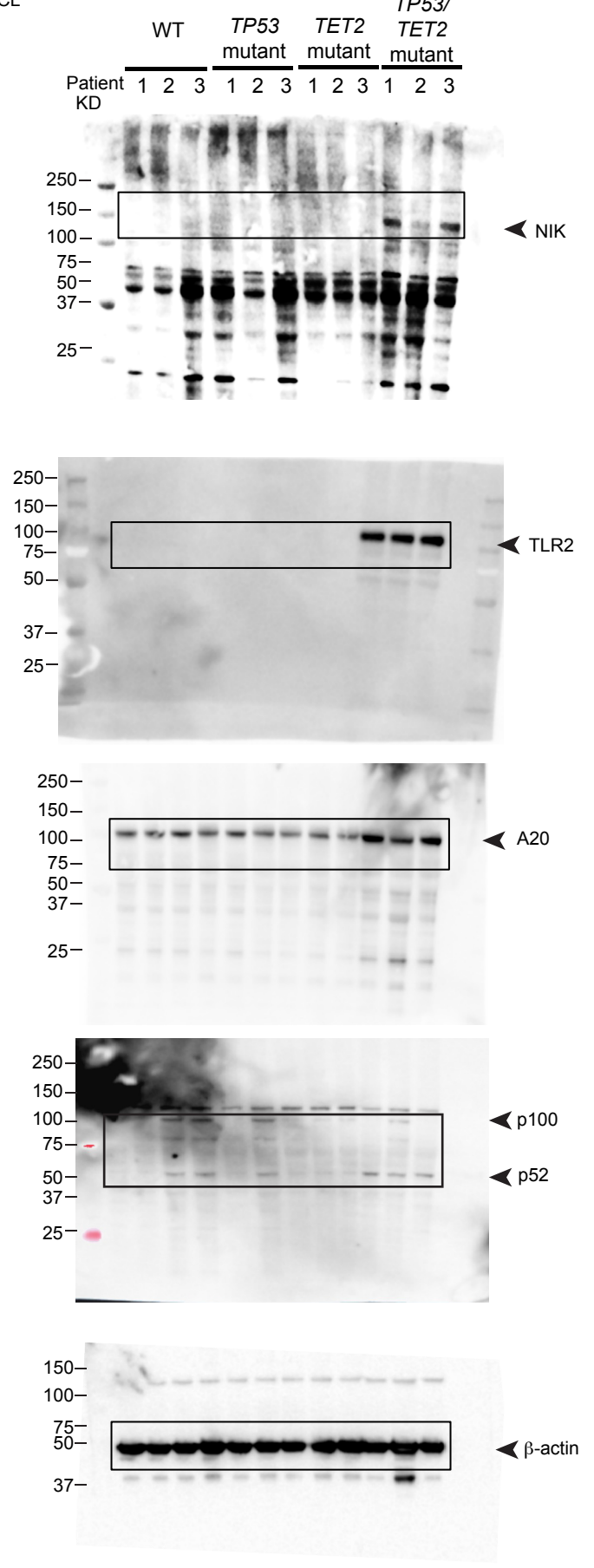

Full unedited blots for Figure 5D

NE

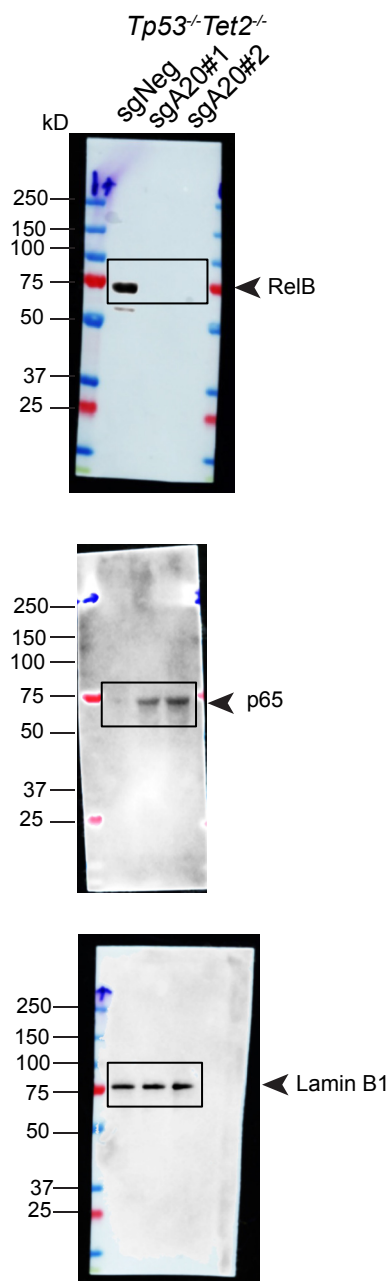

WCL

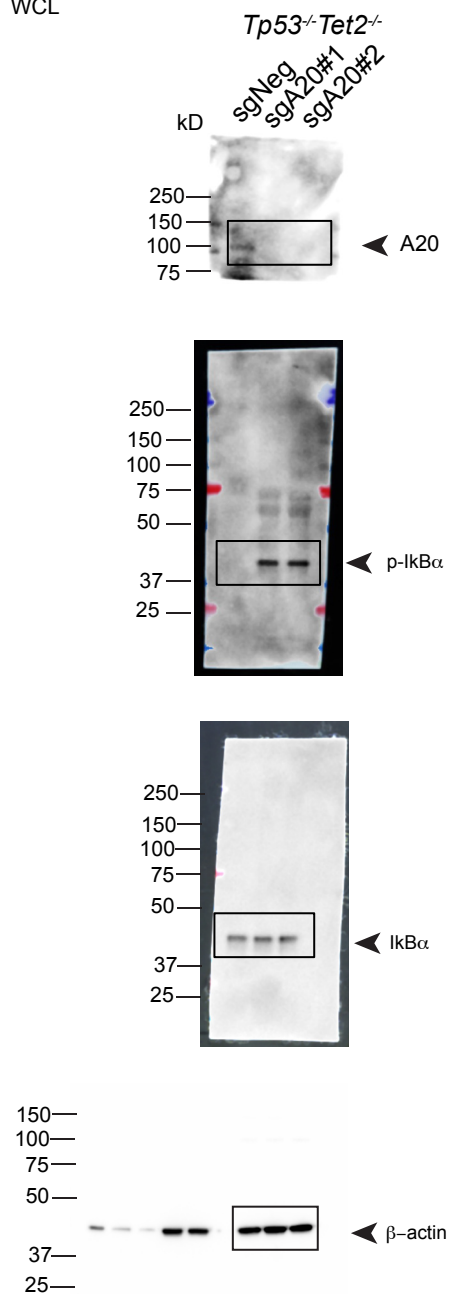

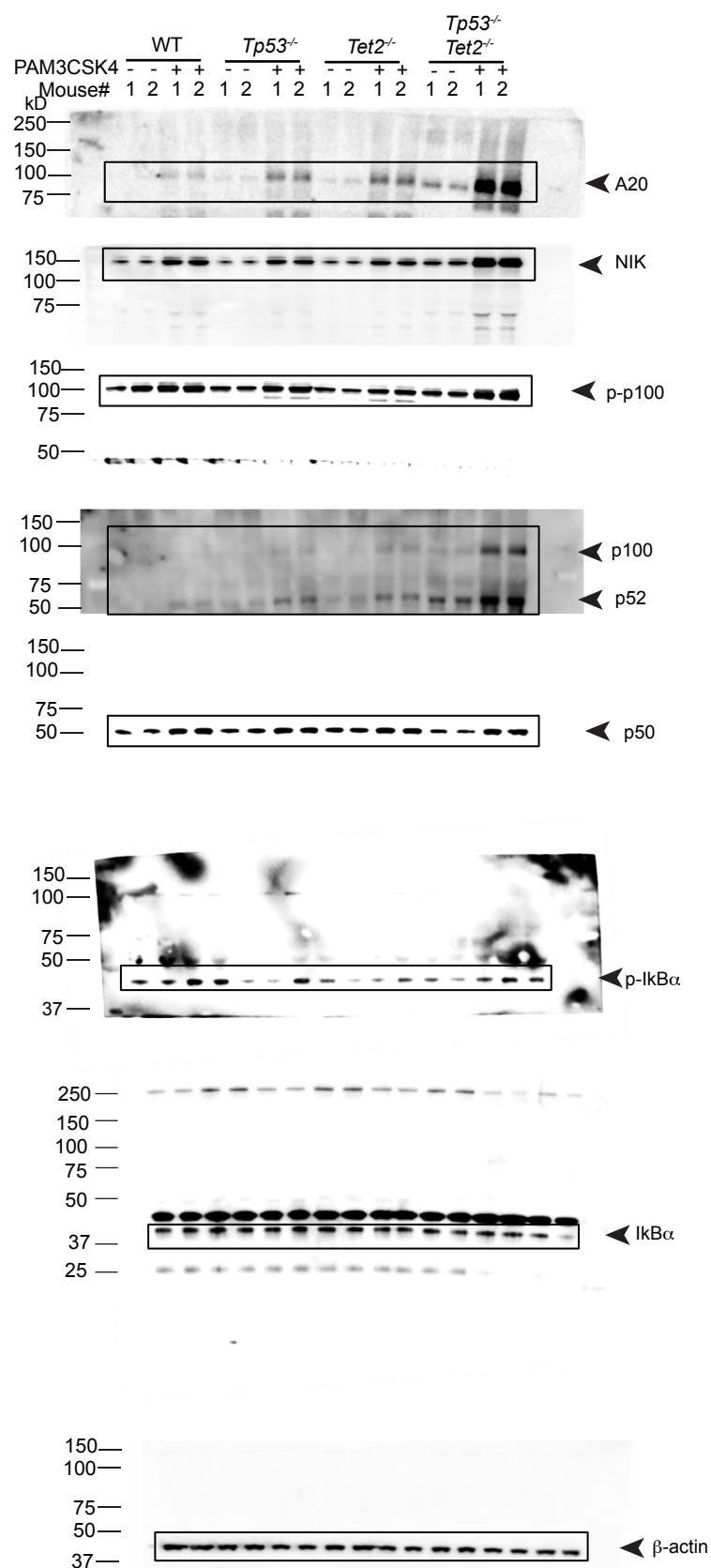

-/-

Supplement: Unedited blot and gel images [file jci-135-184021-s105.pdf]
